# ANKS3 Co-Localises with ANKS6 in Mouse Renal Cilia and Is Associated with Vasopressin Signaling and Apoptosis *In Vivo* in Mice

**DOI:** 10.1371/journal.pone.0136781

**Published:** 2015-09-01

**Authors:** Laure Delestré, Zeineb Bakey, Cécilia Prado, Sigrid Hoffmann, Marie-Thérèse Bihoreau, Brigitte Lelongt, Dominique Gauguier

**Affiliations:** 1 Sorbonne Universities, University Pierre and Marie Curie, University Paris Descartes, Sorbonne Paris Cité, INSERM, UMR_S1138, Cordeliers Research Centre, Paris, France; 2 Sorbonne Universities, University Pierre and Marie Curie, UMR_S1155, Paris, France; 3 INSERM, UMR_S1155 Hôpital Tenon, Paris, France; 4 Medical Research Centre, Medical Faculty Mannheim, University of Heidelberg, Mannheim, Germany; 5 National Genotyping Centre, 2 rue Gaston Crémieux, Evry, France; 6 Institute of Cardiometabolism & Nutrition, Pitié-Salpêtrière Hospital, University Pierre and Marie-Curie, Paris, France; UCL Institute of Child Health, UNITED KINGDOM

## Abstract

Mutations in Ankyrin repeat and sterile alpha motif domain containing 6 (ANKS6) play a causative role in renal cyst formation in the PKD/Mhm(*cy*/+) rat model of polycystic kidney disease and in nephronophthisis in humans. A network of protein partners of ANKS6 is emerging and their functional characterization provides important clues to understand the role of ANKS6 in renal biology and in mechanisms involved in the formation of renal cysts. Following experimental confirmation of interaction between ANKS6and ANKS3 using a Yeast two hybrid system, we demonstrated that binding between the two proteins occurs through their sterile alpha motif (SAM) and that the amino acid 823 in rat ANSK6 is key for this interaction. We further showed their interaction by co-immunoprecipitation and showed *in vivo* in mice that ANKS3 is present in renal cilia. Downregulated expression of Anks3 *in vivo* in mice by Locked Nucleic Acid (LNA) modified antisense oligonucleotides was associated with increased transcription of vasopressin-induced genes, suggesting changes in renal water permeability, and altered transcription of genes encoding proteins involved in cilium structure, apoptosis and cell proliferation. These data provide experimental evidence of ANKS3-ANKS6 direct interaction through their SAM domain and co-localisation in mouse renal cilia, and shed light on molecular mechanisms indirectly mediated by ANKS6 in the mouse kidney, that may be affected by altered ANKS3-ANKS6 interaction. Our results contribute to improved knowledge of the structure and function of the network of proteins interacting with ANKS6, which may represent therapeutic targets in cystic diseases.

## Introduction

Cystic kidney diseases cover a broad range of severe genetic conditions in human, including polycystic kidney disease (PKD) and nephronophthisis, unified by the occurrence of fluid filled cysts primarily in the kidney often associated with extra-renal manifestations. Autosomal dominant PKD (ADPKD) is one of the most common genetic disorders, with an estimated prevalence of 1:400 to 1:1,000 live birth. It is characterized by the development of bilateral renal cysts frequently caused by mutations in proteins *PKD1* and *PKD2* encoding polycystins 1 and 2 [1].Genetic analyses carried out in the laboratory in the PKD/Mhm(*cy*/+) rat model of ADPKD revealed that the disease spontaneously develops as a consequence of a mutation (R823W) in the Sterile Alpha Motif (SAM) domain of Ankyrin repeat and sterile alpha motif domain containing 6 (*Anks6*) [2]. Overexpression of transgenic Anks6^(pR823W)^ in the renal tubular epithelium leads to the development of cystic lesions in the kidney that are largely similar to those described in PKD/Mhm(*cy*/+) rats [3]. Recent results from genetic studies of ANKS6 in humans and *Anks6*gene targeting in model species further underlined the important role of the protein in renal function and cystogenesis [4–7].

The protein ANKS6 contains two types of functional domains: several tandem Ankyrin repeats (8–11 depending on the species) and a SAM domain in its N- and C-terminal extremities, respectively. Even though the presence of these domains suggests a function of ANKS6 in protein binding and scaffolding, identification of interacting partners is essential to integrate the protein in functional networks and understand its biological roles. This line of research has been initiated with the detection of interactions between ANKS6 and the RNA binding protein BICC1, which have shed light on BICC1 mediated mechanisms (cell polarity, cAMP signaling, cilia orientation and cilia-driven-fluid flow) that may be involved in renal cystogenesis when interaction between BICC1 and ANKS6 is affected [7–9]. More recently, localisation of ANKS6 to the primary cilium and interactions between ANKS6 and proteins involved in nephronophthisis (INVS, NHPH3, NEK8) were demonstrated [4, 6, 7]. Protein structure analyses identified the Ankyrin repeats and sterile alpha motif domain containing 3 (ANKS3) as a potential direct binding partner of ANKS6 and provided crystal structures showing that the R823W mutation in ANKS6 destabilizes its SAM domain and prevents ANKS3-ANKS6 interaction [10].

In the present study, we report experimental confirmatory evidence of both direct physical interaction between ANKS6 and ANKS3 through the SAM domain of ANKS6 and demonstrate ANKS3 localisation in renal cilia in mice. We further provide initial characterisation of the functional role of ANKS3 *in vivo* in the regulation of the expression of *Anks6* and genes encoding proteins involved in vasopressin-driven water reabsorption (*Vit32*, aquaporins 1, 2 and 3), in apoptotic and proliferative signaling pathways (caspases 3 and 9, p53) and in cilium structure (*Cep290*, *Gli2*, *Nek8* and *Nphp1*, *2*, *4 and 5)* in the mouse kidney.

## Methods

### Yeast Two Hybrid screening

Plasmid constructions pGBD-B and pACT2-B derive from pGBD-C1 and pACT2, respectively (Table 1) [11]. Bait plasmids were constructed in the PJ69-4A yeast strain by homologous recombination of the coding sequences for the regions of ANKS6 protein containing Ankyrin repeats (1–482), SAM domain (667–885) or the central region (428–766) into pGBD-B. Plasmids were rescued from these yeast clones according to standard protocols and incorporated into *Escherichia Coli* strain DH5α (Life Technologies Corporation, Carlsbad, CA). All inserts were fully sequenced.

**Table 1:** Plasmids used in this study.

Plasmid Name	Vector	Insert	Reference
pGBD-B	pGBD-B		Sample et al. 2005
pGBD-B-Anks6-ANK	pGBD-B	rat Anks6 Ankyrin repeats region (1–481)	This study
pGBD-B-Anks6-MID	pGBD-B	rat Anks6 middle region (428–766)	This study
pGBD-B-Anks6-SAM	pGBD-B	rat Anks6 SAM domain (667–885)	This study
pACT2-B	pACT2-B		Sample et al. 2005
pACT2-B-Prey1	pACT2-B	mouse full length Anks3	This study
pACT2-B-Prey2	pACT2-B	mouse full length Anks3	This study
pACT2-B-Prey3	pACT2-B	mouse full length Anks3	This study
pACT2-B-Prey4	pACT2-B	mouse full length Anks3	This study

To perform the interaction screen, two haploid host strains of opposite mating were used, one (PJ69-4A) containing the bait construct, and the other (Y187) containing the prey cDNA library constructed using mouse 17-day embryo total RNA (Clontech Laboratories Inc., Mountain View, CA) [11]. 1x10^9^ cells of each yeast strains were harvested and mated. The spread cell suspension was plated on a Yeast extract Peptone Adenine Dextrose (YPAD) plate for 5–6 hours at 30°C. Then hybrids were resuspended and 10-fold serial dilutions were plated onto plates lacking leucine or tryptophane or both amino acids in order to evaluate the number of cells screened from the library. The remaining of the yeast suspension was plated onto plates lacking leucine, tryptophane and histidine. Colonies growing in triple drop out medium were tested for growth in drop out medium lacking leucine, tryptophane and adenine in order to confirm interactions between the bait and the preys. Interaction assays were performed to verify screening results. Yeast cells PJ69-4A and PJ69-4α were transformed with bait plasmids (pGBD-B) and the plasmids containing rescued preys (pACT-B), respectively. Then both strains were mated and plated onto plates lacking leucine, tryptophane and adenine and plates lacking leucine, tryptophane and histidine. Interaction studies were carried out after 2–3 days of incubation.

### Yeast strains

The following yeast strains were used in this study.

PJ69-4a: Genotype: *MATa*, *trp1-901*, *leu2-3*.*112*, *ura 3–52*, *his3-200*, *gal4Δ*, *gal80Δ*, *LYS2*::*GAL1-HIS3*, *GAL2-ADE2 met2*::*GAL7-lacZ*

PJ69-4alpha: Genotype: *MATalpha*, *trp1-901*, *leu2-3*.*112*, *ura 3–52*, *his3-200*, *gal4Δ*, *gal80Δ*, *LYS2*::*GAL1-HIS3 GAL2-ADE2*, *met2*::*GAL7-lacZ*

Y187: Genotype: *MATalpha*, *trp1-901*, *leu2-3*.*112*, *ura 3–52*, *his3-200*, *ade2-101*, *gal4Δ*, *met-*, *gal80Δ*, *URA3*::*GAL1UAS-GAL1TATA-lacZ*

### *Anks3* expression inactivation using LNA Antisense Oligonucleotide

A cocktail of 3 specific LNA AntiSense Oligonucleotides (ANKS3 ASO) was used to block *Anks3* gene expression *in vivo* in mice. LNA ASOs complementary to the mouse *Anks3* mRNA were designed on IDT DNA (Integrated DNA Technologies) [12] using procedures already optimised to downregulate gene expression in kidney [13]. The oligodeoxynucleotides are 21-nucleotide long with LNA bases and phosphorothioate linkage modifications to prevent their *in vivo* hydrolysis by nucleases and the inflammatory response and to increase binding efficiency to its target (Eurogentec, Seraing, Belgium) (S1 Table). The LNA ASOs are also conjugated to Alexa Fluor 647 fluorescent dye on amine C7 of the 3’ extremity to control the distribution of the LNA ASOs in mice. Absence of cross reactivity with related sequences in GenBank was verified. A scrambled oligonucleotide (SCR ASO), which has the same length and base composition as the LNA ASOs targeting *Anks3* mRNA but in a random order, was used as control.

Four day old C57Bl/6 mice (Janvier, Saint Berthevin, France) were injected intraperitoneally with saline, or with ANKS3 ASO or SCR ASO (300 pmol/injection) dissolved in saline. Injections were repeated every other day. A total of 6 injections were performed over a period of 14 days. In parallel, an additional control group was injected with saline (group Ctr). Body weight was monitored throughout the injection period (postnatal days 4 to 18). Mice were killed by intraperitoneal injection of pentobarbital 4 days (aged 22 days) and 12 days (aged 30 days) after the last ASO injection. Urine was collected by urinary puncture and plasma was prepared from blood collected by cardiac puncture in EDTA-tubes. Samples were stored at −80°C until assays. Kidney, liver and spleen were dissected and weighed. Half of each sample was immediately snap-frozen in liquid nitrogen and stored at −80°C, and the other half was treated with a 4% phosphate-buffered paraformaldehyde (PFA) solution (pH 7.2) for histology analysis.

All animal studies were authorized by a licence (Ce5/2012/072) under the Charles Darwin Ethics Committee in Animal Experiment, Paris, France. They are compliant with ARRIVE guidelines (**S2 Table**). Mice were housed in the animal research facility at the Cordeliers Research Centre and maintained in a controlled environment (23±2°C; 12 hours light/dark cycles).

### Immunohistochemistry

Kidneys were transversally sectioned, fixed with 4% paraformaldehyde overnight and embedded in paraffin. Antigen was unmasked on 5μm sections either by incubation in citrate buffer (0.01M, pH6.0) or by proteinase K (20μg/ml) treatment, followed by incubation in 3% BSA in PBS. Primary antibodies were diluted according to manufacturer's instructions and applied overnight at 4°C followed by incubation with the second antibodies conjugated to Alexa Fluor (dil 1/1000). When blocking peptide or recombinant protein were available, primary antibodies were incubated with a 50-fold excess of peptide/protein and used in a control section. If they were not available, controls were performed with identical concentrations of IgGs of the same species. Immunohistochemical studies for ANKS3 and ANKS6 were carried out on consecutive 3μm paraffin section using a biotinylated secondary antibody (anti-rabbit, 1:200, Sigma or anti-goat Vector, BA9500, 1:200). Incubation with avidin-biotin complex reagent and colorimetric detection was performed according to the manufacturer’s instruction (Vectastain Elite ABC-Peroxidase Kit, DAB Substrate Kit, Vector Laboratories, Burlingame, CA).

### Assessment of apoptosis and proliferation

Apoptotic cells were detected by morphologic criteria including cell shrinking, formation of apoptotic bodies and condensation of chromatin, as well as by in situ TdT mediated dUTP Nick End Labeling (TUNEL) method. Kidneys were fixed in 4% PFA, embedded in paraffin and sections (3μm) were prepared. Immunofluorescence (FITC) TUNEL technique was performed according to the manufacturer’s instruction (Apoptag, QBiogene, Irvine, CA). Kidney sections were counterstained with an antibody against PCNA conjugated to HRP to detect proliferative cells. Peroxidase was revealed by incubation with its substrate 3-amino-9-ethyl-carbazole (AEC). Slides were then counterstained with DAPI (2μg/ml), mounted in Tris glycerol buffer and observed under microscope (Carl Zeiss SA, Le Pecq, France). The percentage of apoptotic and proliferative nuclei was determined on 6 non consecutive microphotographs randomly taken for 4 SCR and 4 ANKS3 ASO mice, 4 days and 12 days after the last injection. Apoptotic and normal nuclei were counted by two independent examiners unaware of the identity of the mice.

### Renal biological parameters

Urea and proteinuria were assayed on a Konelab20 automate (Thermo Scientific, Waltham, MA) using the pyrogallol red method (proteinuria). Plasma urea nitrogen was assessed using a colorimetric detection kit (Arbor Assays, Ann Arbor, MI).

### DNA sequencing

Sequencing reactions were performed with BigDye terminators, Version 3.1, and analyzed by ABI PRISM 3700 DNA Analyser (Applied Biosystems, Foster City, CA). Nucleotide sequence databases were searched for homologous sequences by BLAST search analysis.

### RNA isolation and reverse-transcription PCR (RT-PCR)

Total RNA was prepared from frozen tissues using the RNeasy Mini kits (Qiagen, GmbH, Hilden, Germany). RNA amount and quality were evaluated using a Nanodrop (Thermo Scientific, Waltham, MA). The cDNA templates for RT-PCR and Q-RT–PCR were prepared by random priming of 2 μg of total RNA with 200 units of Moloney Murine Leukemia Virus reverse transcriptase.

Quantification of *Anks3*, *Anks6*, *Aqp1*, *Aqp2*, *Aqp3*, *Cep290*, *Gli2*, *Icam1*, *Nek8*, *Nphp1*, *Nphp4*, *p53* and *Vit32* expression was carried out using the SYBRGreen technology (Qiagen, GmbH, Hilden, Germany) using oligonucleotides listed in S3 Table. Due to the controversial use of *Actb* gene as an endogenous internal control for RT-PCR analyses, quantity and quality of the cDNA templates were evaluated by analysis of *Actb* mRNA and confirmed by analysis of *Gusb* mRNA [14]. Quantitative PCR were performed on a Rotor Gene 3000 real time PCR platform (Corbett Research Ltd, Qiagen, GmbH, Hilden, Germany). PCR reactions were performed as previously described [15] using Rotor-Gene SYBR Green RT-PCR kits (Qiagen, GmbH, Hilden, Germany).

### Western Blot analysis

Whole tissue extracts were prepared from kidney for western blot analyses. Frozen samples (25–30 mg) were homogenised in a lysis buffer supplemented with phosphatase and protease inhibitors cocktail (Thermo Fisher Scientific, Rockford, IL) using a Tissue Lyser mechanical homogenizer (Qiagen, GmbH, Hilden, Germany). Cellular debris were pelleted by centrifugation and protein concentration was determined from the supernatant using a Bradford assay. 50 μg of kidney lysate proteins were resolved on a 10% SDS-polyacrylamide gel under reducing conditions and transferred onto nitrocellulose filters using Mini Trans-Blot Electrophoretic Transfer Cell (Bio-Rad Laboratories GmbH, Munich, Germany). Membranes were blocked in 5% powdered milk in TBS-Tween (TBST 0.2%) and incubated with relevant primary antibodies in TBST 0.2% (rabbit anti-Caspase 3, rabbit anti-Caspase 9, rabbit anti-ANKS3, goat anti-Anks6, rabbit anti-AQP2 or rabbit anti-beta actin antibody). Membranes were washed extensively with TBST 0.2%, incubated either with 1:5000 anti-rabbit-HRP or anti-goat-HRP, and proteins were visualized with enhanced chemiluminescence prime reagents according to the manufacturer’s instructions (GE Healthcare Europe, GmbH, Munich, Germany) or with corresponding IgG conjugated to alkaline phosphatase (0.02 μg/ml). Alkaline phosphatase activity was revealed by adding the NBT/BCIP substrate (nitro blue tetrazolium/5-bromo-4-chloro-3-indolyl phosphate complex in 100 mM Tris-HCl, 100 mM NaCl and 5 mM MgCl2, pH 9.5). The reaction was stopped in 20 mM Tris-HCl, 5 mM EDTA, pH8.0. Immunoblot images were acquired in a Bio-Imaging Systems MF-ChemiBIS 4.2 (DNR Bio-Imaging Systems, Jerusalem, Israel). Densitometric analysis on ImageJ (U.S. National Institutes of Health, Bethesda, MD) was performed for quantification.

### Co-immunoprecipitation

Immunoprecipitations were performed according to the manufacturer’s procedure (Miltenyi Biotec, Bergisch Gladbach, Germany). Briefly, 750 μg of protein extracts were incubated with rabbit anti-Anks3 (1μg) or goat anti Anks6 (2μg) antibody or control IgGs from the same species overnight at 4°C. The immune complexes were then linked to Protein A or Protein G conjugated to microbeads for 1 hour at 4°C followed by immunopurification using MAC μcolumns. Eluted proteins were used for Western Blot analysis.

### Antibodies and reagents

Mouse monoclonal antibody directed against Proliferating Cell Nuclear Antigen (PCNA, clone PC10) conjugated to horseradish peroxidase (HRP) was obtained from Dako Cytomation SAS (Glostrup, Denmark). Goat polyclonal antibodies against AQP2, ANKS6 and ANKS3 and rabbit polyclonal antibody against AVPR2 were obtained from Santa Cruz (Santa Cruz Biotechnology, Santa Cruz, CA). Rabbit polyclonal antibodies against ANKS3 and rabbit monoclonal antibody against Caspase 3 were purchased from Novus (Novus Biologicals, Littleton, CO) and from BD Pharmingen (BD, Franklin Lakes, NJ), respectively. Rabbit polyclonal antibody against Caspase 9 and secondary anti-goat antibody coupled to HRP were purchased from Abcam (Abcam PLC, Cambridge, UK). Secondary anti-rabbit antibody conjugated to HRP was purchased from Cell Signaling Technology (Cell Signaling Technology Inc., Danvers, MA). Secondary antibodies conjugated to Alexa were purchased from Invitrogen (Invitrogen, Eugene, OR).

### Statistical analysis

Statistical analyses were performed with non-parametric Mann- Whitney U test or one way ANOVA test. A p value of <0.05 was considered statistically significant.

## Results

### ANKS3 and Anks6 interact via their SAM domain

**Fig 1:**
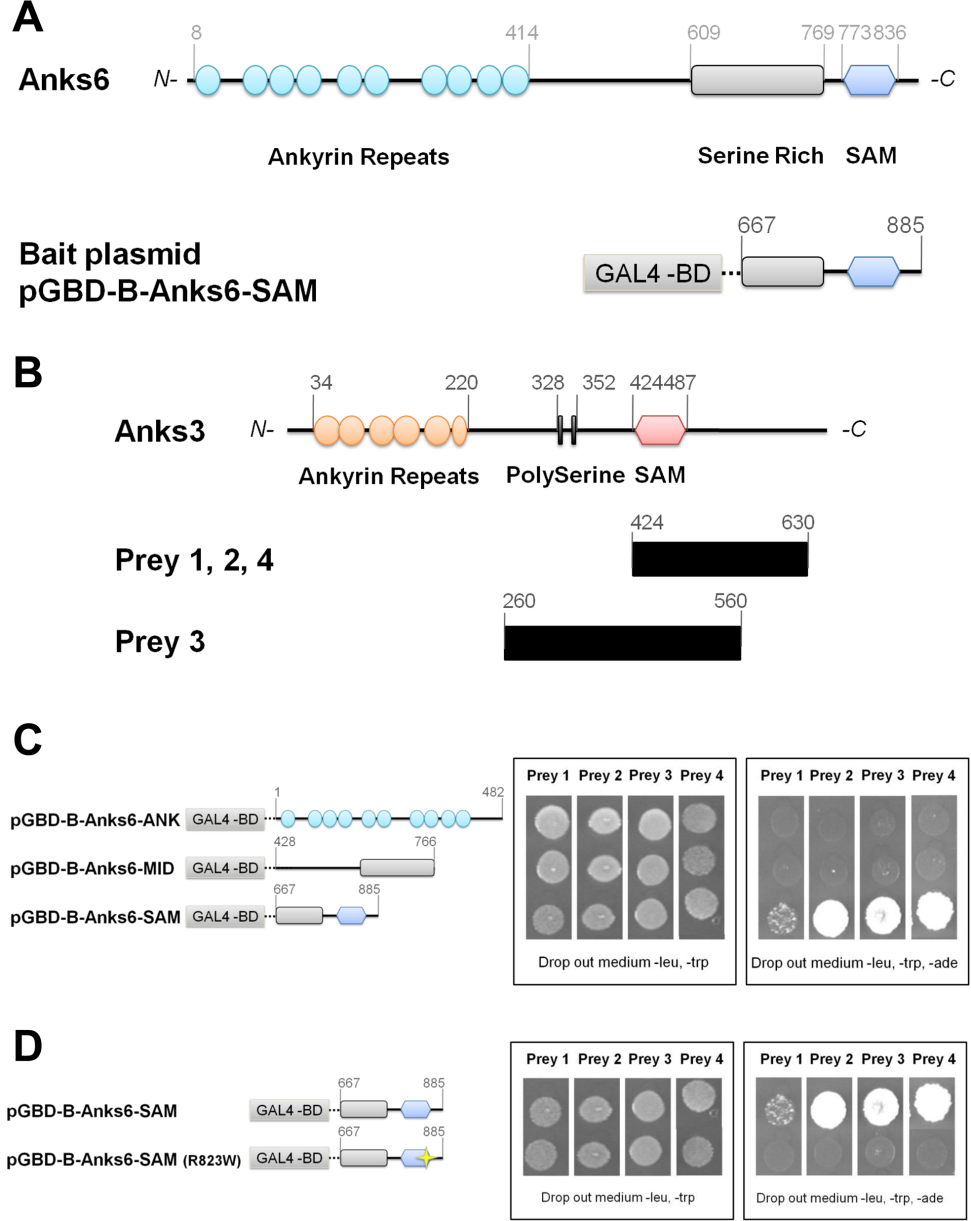
Identification of ANKS3, a partner of ANKS6. (**A**) Schematic representation of ANKS6 protein and the bait construct (pGBD-B-Anks6-SAM) used to perform the yeast two hybrid screen. (**B**) Schematic representation of ANKS3 protein for comparison with cDNA sequences of prey 1, 2, 3 and 4, recovered from the yeast two hybrid screen. (**C**) Interaction of ANKS6 with the 4 preys in interaction assays in yeast. The bait constructions containing either the Ankyrin repeats region of ANKS6 (pGBD-B-Anks6-ANK), or the Serine Rich region (pGBD-B-Anks6-MID), or the SAM domain (pGBD-B-Anks6-SAM) are shown on the left. The ability of these domains to interact with the preys is shown on the right. (**D**) Effect of the mutation R823W in the SAM domain of ANKS6 on the interaction between the preys and ANKS6. The construction pGBD-ANKS6-SAM and its mutated version (pGBD-ANKS6-SAM-R823W) are shown on the left. The ability of the SAM and SAM-R823W domains of the ANKS6 protein to interact with the preys is shown on the right.

To identify protein partners of ANKS6, we used a yeast two hybrid system and screened a mouse 17-day embryo cDNA library. The screen was performed using the C-terminal extremity of the rat protein Anks6 harboring the SAM domain as the bait protein, which was expressed from the pGBD-B plasmid (Fig 1A). From the mating of 1x10^9^ bait and 1x10^9^ prey transformants, 384 hybrids were obtained and 25 were the result of positive protein interaction. From these, 4 clones were fully sequenced. All of them corresponded to partial cDNA sequences of the geneAnks3. The predicted ANKS3 protein is 655 aa long and presents organization of domains similar to that of ANKS6 (Fig 1B). Three of the four preys encoded the C-terminal extremity of ANKS3 and contained the SAM domain (aa 424–630) (Preys 1, 2, 4). The fourth sequence encoded the central region of ANKS3 containing the Poly Serine region and the SAM domain (aa 260–560) (Prey 3) (Fig 1B).

To determine the regions of ANKS6 that are important for the interaction with ANKS3, the four preys were retransformed into yeast and interaction assays with domains of ANKS6 were performed (Fig 1C). We showed that neither the Ankyrin repeats region nor the serine rich region of ANKS6 interacts with the central region and the C-terminal extremity of ANKS3 (Fig 1C). In contrast, the C-terminal extremity of ANKS3 binds the SAM domain of ANKS6. This interaction was disrupted by the replacement of the arginine at position 823 by a tryptophan, which corresponds to the amino acid change causing PKD in the PKD/Mhm(*cy*/+) rat (Fig 1D). These results provide experimental confirmation that ANKS3 is a direct protein partner of ANKS6, which bind through their SAM domains and that the amino acid at position 823 of the SAM domain of ANKS6 is essential for the interaction to occur.

### ANKS3 and ANKS6 show extensive sequence and structural similarities

To evaluate structural and functional relationships between ANKS6 and ANKS3 that may account for the mechanism of interactions between the two proteins, we compared the amino acid sequence of the proteins in five species (human, rat, mouse, chicken and zebrafish) (S1 Fig). Multiple sequence alignment analysis showed that ANKS3 is weakly similar to ANKS6 in all five species when sequences of the whole proteins were considered. However, when analyses were performed for the ANKS and SAM protein regions independently, sequence conservation between ANKS3 and ANKS6 across the five species was only 26% for the ANKS repeats regions (S1A Fig) and 86% for the SAM domains (S1B Fig). Such extensive similarities in the sequence of the SAM domains in distant phylogenetic species strongly suggest that the SAM domain of ANKS6, where the mutation is located in the PKD/Mhm(*cy*/+) rat, is critical for its binding to ANKS3and their biological function.

### ANKS3 is present in the mouse kidney

**Fig 2:**
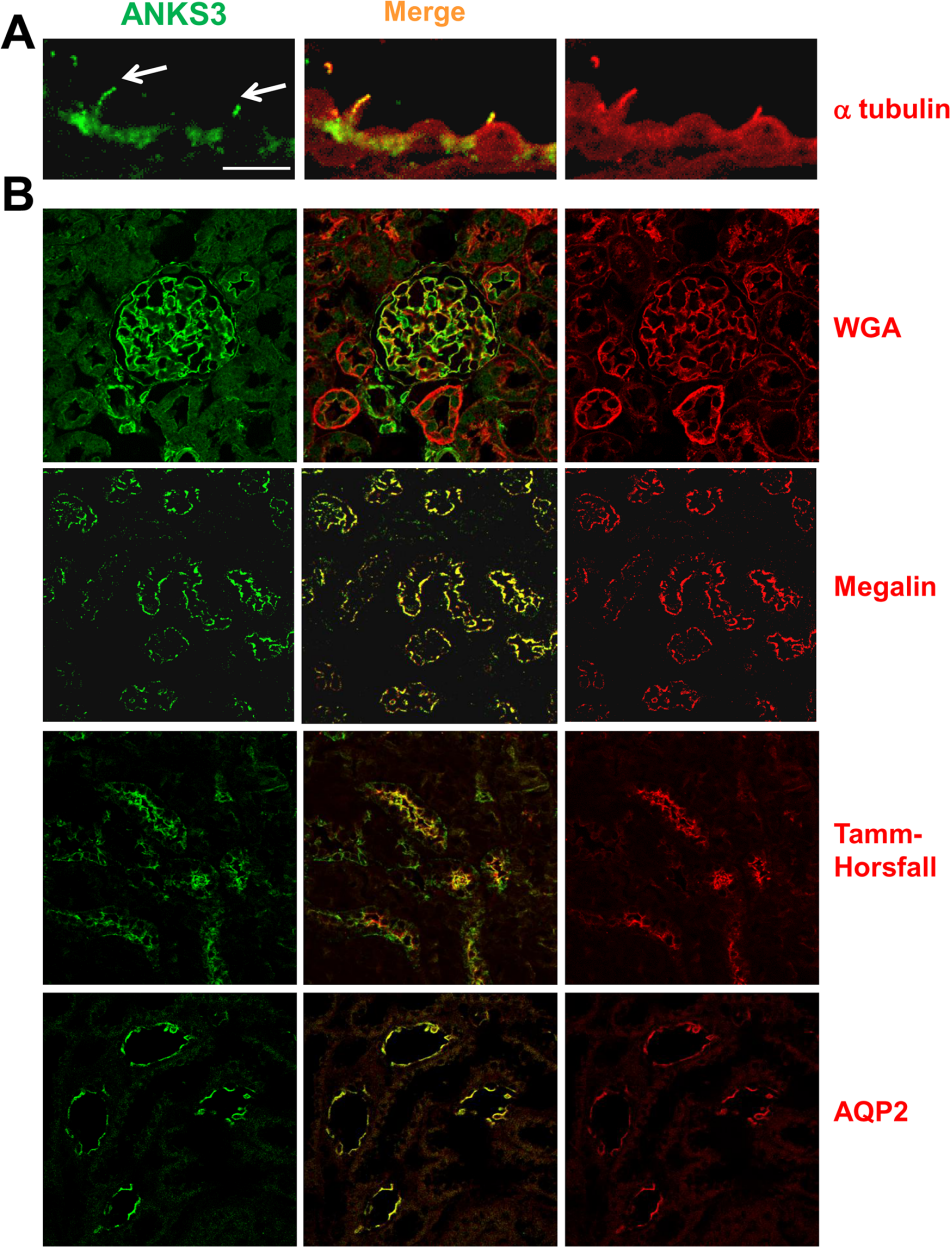
Expression of ANKS3 in kidney of control mice. (A) ANKS3 expression revealed by Alexa Fluor 488 in green (left column) in a representative cilium stained with acetylated α-tubulin revealed by Alexa Fluor 594 in red (right column); scale bar: 3.5μm. (B) Representative photographs of 6 adult kidneys. ANKS3 staining (revealed by Alexa Fluor 594 in green, left column) co-localises (right column and merge staining in central column) with WGA lectin conjugated to TRITC in red, a marker of podocyte in glomeruli, (scale bar: 90μm) and in tubules stained with Megalin (proximal tubule marker), Tamm-Horsfall protein (Thick ascending limb of Henle’s loop marker) and with AQP2 (collecting duct marker); scale bar: 300μm.

We initially investigated the cellular location of ANKS3 by immunohistochemistry on mouse renal sections. The protein encoded by *Anks3*was observed in tubular cilia where it co-localises with acetylated α-tubulin (Fig 2A). ANKS3 staining was observed on the whole length of cilia, with a punctuated expression pattern. ANKS3 was also present in glomeruli stained with WGA and at the apical side of tubules (Fig 2B). Double labelling experiments with megalin, Tamm Horsfall protein (TH) and aquaporin 2 (AQP2) showed that ANKS3 is present in the proximal tubules, the thick ascending limb of Henle's loop (TAL) and in the collecting ducts.

### ANKS3 and ANKS6 interact in kidney in mice

**Fig 3:**
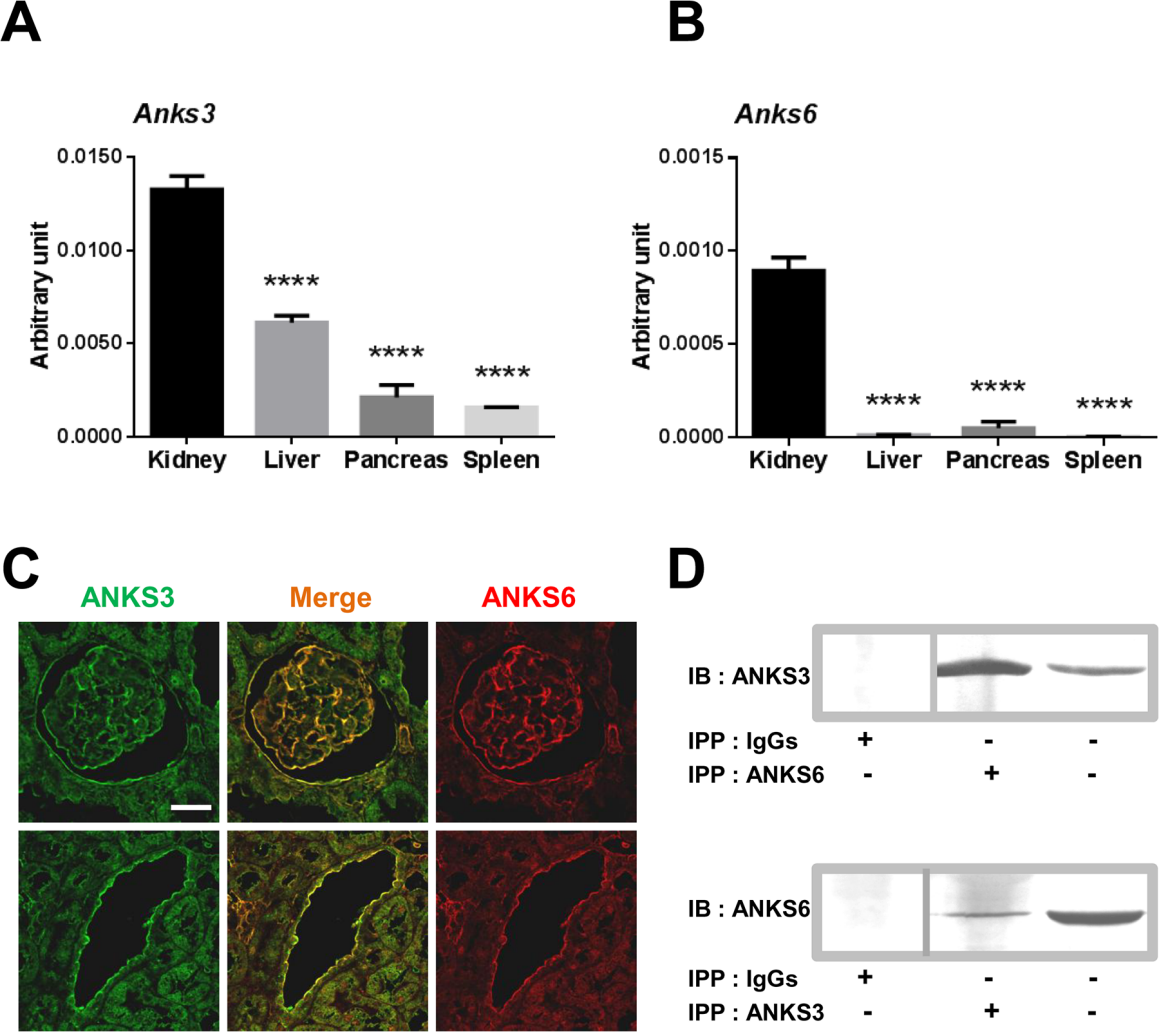
Organ expression, physiological interaction and renal location of ANKS3 and ANKS6 in the mouse. ANKS3 and ANKS6 are strongly expressed in the mouse kidney.*Anks3* (**A**) and *Anks6* (**B**) mRNA expression in kidney, liver, pancreas and spleen was evaluated by quantitative RT-PCR. Data are shown as means ± SEM. Quantification of cDNA from 3 mice was normalized to *Actb* gene expression level. One-way ANOVA was applied to assess statistical significance. P<0.0001 significantly different to renal expression of *Anks3* and *Anks6*. (**C**) Co-localisation of ANKS3 and ANKS6 in the kidney. Representative photomicrographs of 6 kidney sections. ANKS3 staining (revealed by Alexa Fluor 488 in green, left column) co-localises (merge staining in central column) with ANKS6 (right vertical column, revealed by Alexa Fluor 594 in red) in glomeruli (upper panel, scale bar: 90μm) and in tubules (lower panel, scale bar: 300μm. (**D**) Immunoprecipitation experiments performed with goat anti-ANKS6 antibody, goat anti-ANKS3 antibody or goat IgGs on protein lysates and followed by an immunoblot performed with rabbit anti-ANKS3 antibody (top) and rabbit anti-ANKS6 antibody (bottom). Note that ANKS6 antibody co-immunoprecipitates ANKS3 in kidney protein lysates.

To test tissue-specific expression of ANKS3 and ANKS6, the relative abundance of the transcripts encoding the proteins was determined in mouse organs where occurrence of cysts is described in PKD. Results from real time quantitative PCR revealed predominant renal expression of both *Anks3* and *Anks6* (Fig 3A and 3B). *Anks3* mRNA levels were higher in the kidney than in the liver (x2.2), pancreas (x6.2) and spleen (x8.5) (P<0.001) (Fig 3A). *Anks6* transcripts were also more abundant in the kidney than in the liver (x71.1), pancreas (x17.5) and spleen (x177.6) (P<0.001) (Fig 3B).

**Fig 4:**
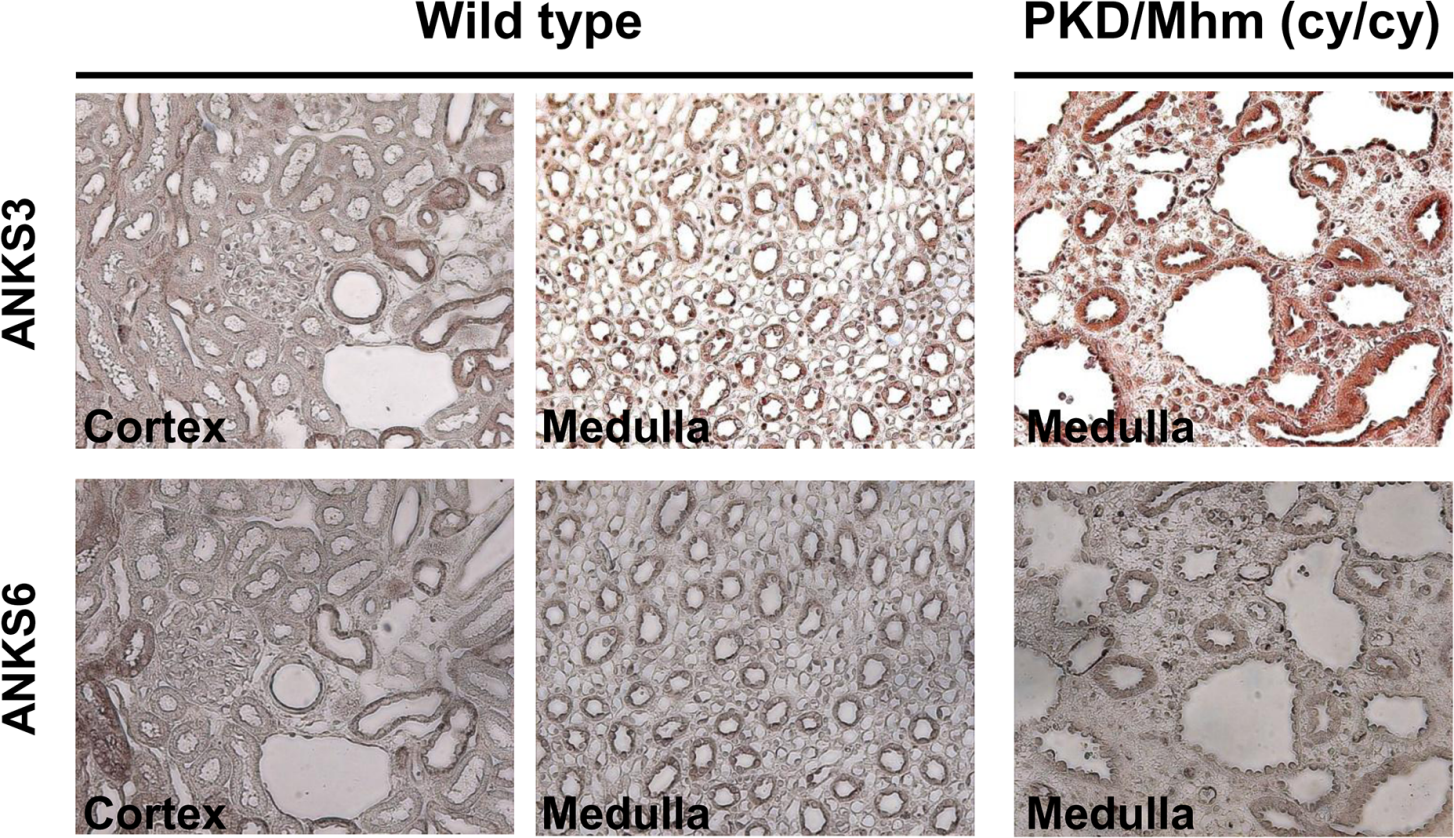
The mutation (R823W) in PKD/Mhm(*cy/cy*) rats does not alter ANKS6-ANKS3 renal co-localisation. Immunohistochemical staining of renal cortex and medulla was performed in 3 week-old wild-type and PKD/Mhm(*cy/cy*) rats for ANKS3 (upper panel) and ANKS6 (lower panel).

Subsequent immunohistological studies in mouse kidneys using specific markers of nephron segments revealed co-localisation of ANKS6 and ANKS3 in glomeruli and in tubules (Fig 3C). Direct interaction between the two proteins in the kidney was confirmed by co-immunoprecipitation of ANKS3 and ANKS6 on kidney lysates with immobilized anti-ANKS3 or anti-ANKS6 antibody (Fig 3D). Finally, we analysed the effect of the R823W mutation in ANKS6 on renal localisation of ANKS3 and ANKS6 and showed that the two proteins remain present in collecting duct cells of renal medulla in both wild-type and *cy/cy* rats (Fig 4).

Our data demonstrate predominant expression of ANKS3 in the kidney and its co-localisation and interaction with ANKS6. We show that the R823W mutation in ANKS6which disrupts the interaction of ANKS6 with ANKS3*in vitro* in our yeast experiments and causes ADPKD in the PKD/Mhm(*cy/cy*) rat, does not alter renal localisation of these proteins.

### Downregulated expression of *Anks3 in vivo* in mice does not alter renal expression of *Anks6*

**Fig 5:**
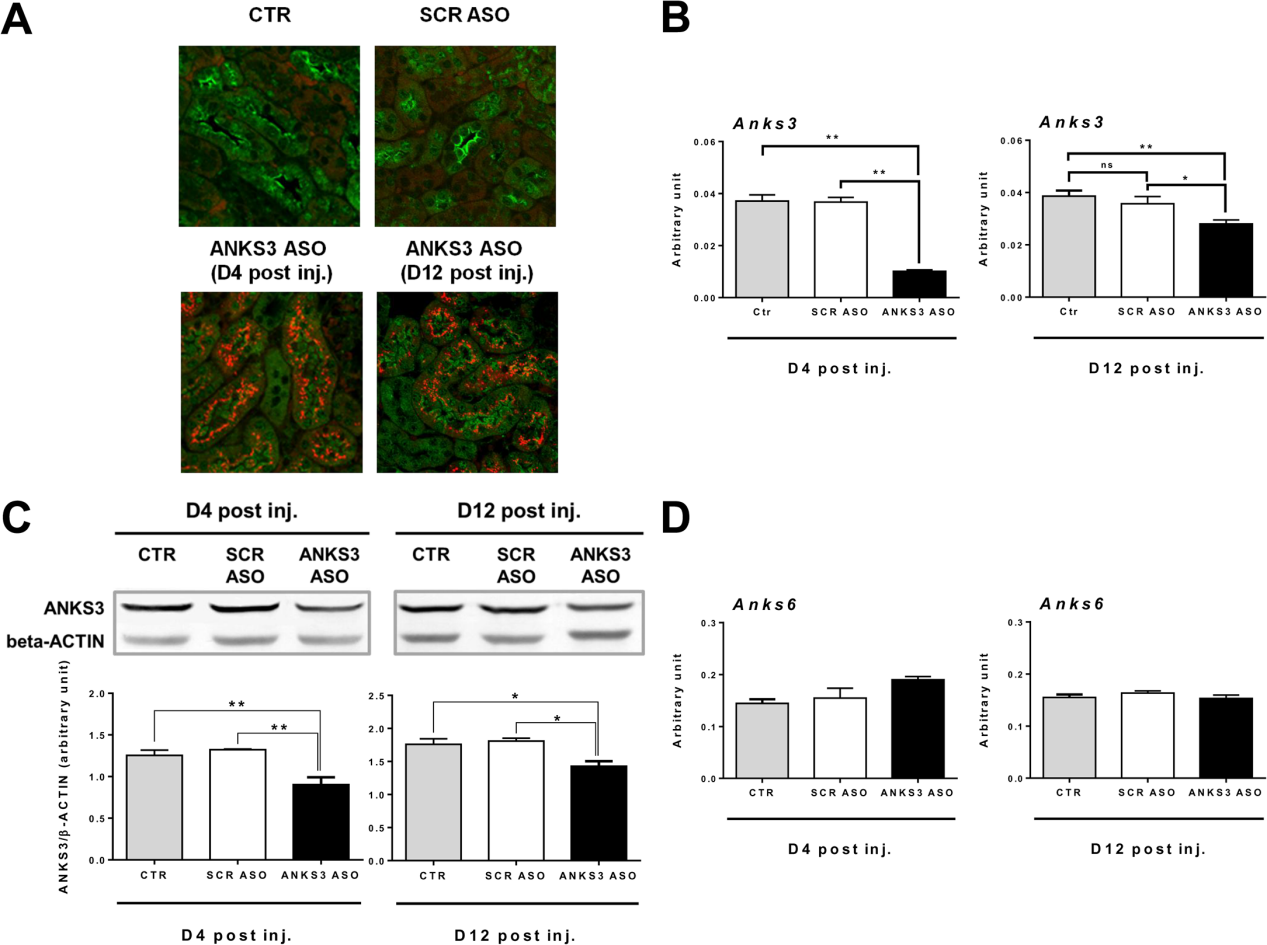
*In vivo* LNA ASO-induced downregulation of*Anks3* expression in mouse kidney. (**A**) Presence of ANKS3 in kidneys treated with saline (CTR) and scrambled AntiSense Oligonucleotides (SCR ASO) 12 days post injection and with ANKS3 Locked Nucleic Acid modified AntiSense Oligonucleotides (ANKS3 LNA ASOs). Representative photomicrographs of kidney sections from 5 mice four and twelve days after the last injection of LNA ASOs (D4 post inj., D12 post inj.). Kidneys were stained with ANKS3 (revealed by Alexa Fluor 488 in green) and the LNA ASOs were localised with the Alexa Fluor 647 marker in red. (**B-D**) *Anks3* renal decreased expression does not affect expression of *Anks6*. Abundance of *Anks3* transcripts (**B**) and protein (**C**), and *Anks6* transcripts (**D**) were evaluated in mouse kidneys four days (D4 post inj., n = 3) and twelve days (D12 post inj., n = 5) after the final injection. cDNA quantification was performed in duplicate and normalized to *Actb* gene expression level. (**C**) Western blots (upper panel) and quantitative protein analysis (lower panel) performed with extracts from kidneys of 5 mice treated with saline (Ctr), scramble ASO (SCR ASO) or ANKS3 ASO four and twelve days after the last injection showed that expression of ANKS3 normalized to that of β-actin is significantly decreased in mice treated with ANKS3 ASO. Data are mean ± SEM. Non-parametric Mann-Whitney U test was used to assess differences between ANKS3 ASO and SCR ASO treated mice.**P<0.01; *P<0.05 significantly different to control mice treated with saline (Ctr) or SCR ASO.

In rodents, only 20% of nephrons are present at birth and the nephrogenesis occurs from E11 to postnatal day 7 (PN7) [16]. To study the *in vivo* function of ANKS3 in the kidney, we tested the biological consequences of *Anks3* downregulated expression induced by Locked Nucleic Acid modified Antisense Oligonucleotides (LNA ASO) in newborn mice. Confocal microscopy analysis revealed the presence of LNA ASO in the kidney of both 22- and 30-days old mice (i.e. 4 and 12 days after the last injection) and the co-localisation of ANKS3 and ANKS3 ASO in the same tubules (Fig 5A). Four days after the final injection of LNA ASOs, renal abundance of *Anks3* transcripts was significantly reduced by over 70% in ANKS3 ASO mice when compared to control mice treated with either saline or a scrambled oligonucleotide (SCR ASO) (Fig 5B). Even 12 days after the final injection of LNA ASOs, renal *Anks3* mRNA levels remained significantly lower in ANKS3 ASO mice than in saline- and SCR ASO-treated mice (-22%). Reduced amount of ANKS3 transcripts in ANKS3 ASO mice was paralleled by significant reduction in abundance of ANKS3 protein when compared to mice treated with saline or SCR ASO (up to -20%; P<0.02), which confirms that significant inhibition of ANKS3 expression *in vivo* was achieved and was specific to LNA ASO treatment (Fig 5C).

To test possible *in vivo* toxicity of LNA ASO administration we determined renal transcript abundance of *Icam1* encoding one of the most important adhesion proteins expressed in endothelial cells, which regulate adhesion and migration of neutrophils. Renal expression of *Icam1* was unaffected in ANKS3 ASO mice4 and 12 days after the last injection when compared to SCR ASO treated controls (data not shown), which rules out presence of kidney inflammatory response to LNA ASOs-mediated *Anks3* mRNA knock down.

Finally, we analysed *Anks6* expression in mouse kidney 4 and 12days after the final injection of LNA ASO. We did not find evidence of significant changes in mRNA level of *Anks6* between the ANKS3 ASO, SCR ASO and saline treated groups (Fig 5D). Therefore, downregulated expression of ANKS3 *in vivo* in the kidney does not significantly affect *Anks6* expression.

### Inhibition of *Anks3* expression *in vivo* affects molecular mechanisms related to water transport in kidney

To investigate the biological consequences of *in vivo Anks3* inactivation we initially determined body growth and variables relevant to renal physiology. During the two weeks of LNA ASO administration, ANKS3 ASO injected mice did not exhibit significant differences in body weight gain compared to the SCR ASO group (S2A Fig). Four and twelve days after final injection, BMI and organ-to-body weight ratios of kidneys, spleen and liver were similar in ANKS3 ASO and SCR ASO groups (S2B–S2F Fig). Renal function evaluated by plasma urea concentration was not altered in ANKS3 ASO mice 4 and 12 days after the final injection compared to controls (S2G Fig).

**Fig 6:**
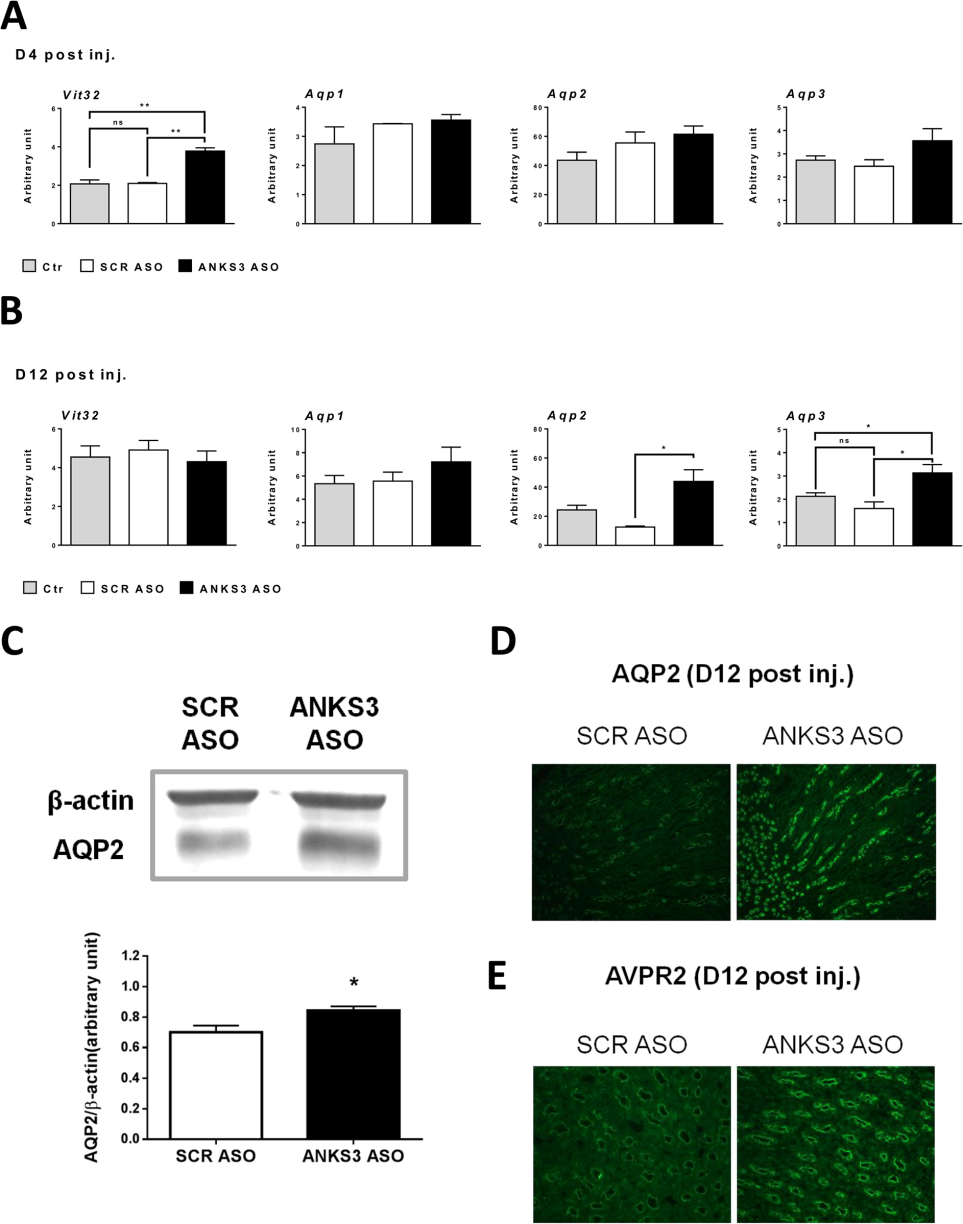
*Anks3* expression knock-down *in vivo* stimulates expression of genes involved in the vasopressin signaling pathway. (**A**-**B**) Renal mRNA expression of *Vit32*, *Aqp1*, *Aqp2* and *Aqp3* was evaluated by quantitative RT-PCR in mice four days (n = 3)(**A**) and twelve days (n = 5)(**B**) after the final injection of Locked Nucleic Acid modified AntiSense Oligonucleotides (LNA ASO). Quantification of each cDNA was performed in duplicate and normalized to *Gusb* gene expression level. (**C**) AQP2 expression in kidney tissues. Representative Western blots performed with protein extracts from 5 kidneys treated with scrambled (SCR) or ANKS3 ASO (top) and quantitative analysis of blots normalized to β-actin expression (bottom) show that AQP2 expression is significantly increased in mice twelve days after the final injection of ANKS3 ASO when compared to mice injected with SCR ASO. (**D**, **E**) Immunofluorescence staining of the papilla for AQP2 (**D**) and AVPR2 (**E**) show a dramatic increase of the proteins in kidneys of ANKS3 ASO treated mice twelve days after the final injection compared to SCR ASO controls. Scale bar: 300 μm. Data are means ± SEM. Non-parametric Mann-Whitney U test was used to assess differences between ANKS3 ASO and control mice injected with saline (Ctr)- or SCR ASO. *P<0.05; **P<0.01 significantly different to controls.

Renal excretion of water is regulated by the peptide hormone vasopressin, which controls vesicular trafficking of aquaporin 2 and renal expression of *Vit32*, *Aqp2* and *Aqp3*. As plasma levels of vasopressin were undetectable in the mouse groups, we determined expression level of the genes encoding *Vit32*, *Aqp1*, *Aqp2* and *Aqp3* as a proxy to assess the regulation of water excretion by vasopressin in *Anks3* knock down mice. Renal expression of *Vit32* was significantly increased (x1.7; p<0.01) 4 days after the last injection in ANKS3 ASO mice when compared to control mice treated with saline or SCR ASO (Fig 6A). Expression of *Aqp1*, *Aqp2* and *Aqp3* was also increased in ANKS3 ASO treated mice (up to 1.4 fold), but differences to the control groups were not statistically significant (Fig 6A). For all four genes, transcript abundance was similar in control mice injected with saline and SCR ASO. This pattern of gene expression in control mice was conserved twelve days after treatment, with the exception of *Aqp2*, which was significantly more elevated in saline injected mice than in SCR ASO treated mice (Fig 6B). Twelve days after LNA ASO injection, elevated renal expression of *Aqp1* (x1.3), *Aqp2* (x4.0; p<0.01) and *Aqp3* (x1.9; p<0.05) was observed in ANKS3 ASO mice when compared to SCR ASO treated controls (Fig 6B). Increased *Aqp2* transcript level in response to ANKS3 ASO treatment was paralleled by a significant increase in AQP2 protein level when compared to SCR ASO controls (Fig 6C). Immunofluorescence histology analysis confirmed the striking increase of aquaporin 2 and arginine vasopressin receptor 2 expression at the protein level in mice injected with ANKS3 ASO (Fig 6D and 6E).

These results suggest that *Anks3* expression knock down *in vivo* does not significantly deteriorate general body condition but is associated with changes in transcription of aquaporin genes suggesting altered regulation of water excretion by vasopressin in the kidney. Differential gene expression regulation patterns 4 and 12 days after LNA ASO injection suggest the involvement of different molecular mechanisms of acute adaptation to *Anks3* knock-down and recovery from the treatment.

### Downregulated expression of *Anks3 in vivo* is associated with altered renal expression of genes encoding primary cilia proteins

Due to the localisation of both ANKS3 and ANKS6 to the primary cilium in the mouse kidney [7]and in Zebrafish [17], and experimental evidence ofANKS3 interaction with nephronophthisis proteins [4, 17], we tested the effects of *in vivo Anks3* knock down on renal expression of genes encoding proteins present in the basal body (*Cep290*, *Nphp5*) and the transition zone (*Nphp1*, *Nphp2*, *Nphp4*) of the primary cilium, and in the ciliary axoneme (*Nek8*, *Gli2*) [18, 19](S3 Fig). Four days after the last injection of LNA ASO, *Nek8* mRNA level was increased in kidney of ANKS3 ASO treated mice, but this effect was statistically significant only when compared to SCR ASO controls. In contrast *Gli2* expression was significantly decreased in ANKS3 ASO mice when compared to both saline and SCR ASO treated controls. The effects on *Gli2* expression were confirmed 12 days after the last injection of LNA ASO and were associated with enhanced expression of*Cep290*. Transcription of *Nphp1*, *Nphp2*, *Nphp4* and *Nphp5* was unaffected by *Anks3* downregulated expression (S3 Fig).

These data indicate that the expression of several genes encoding ciliary proteins in mouse kidney is altered by acute repressed renal expression of *Anks3 in vivo*, suggesting an involvement of this protein in the regulation of ciliary mechanisms.

### *Anks3* downregulated expression in mice affects apoptosis and cell proliferation in kidney

**Fig 7:**
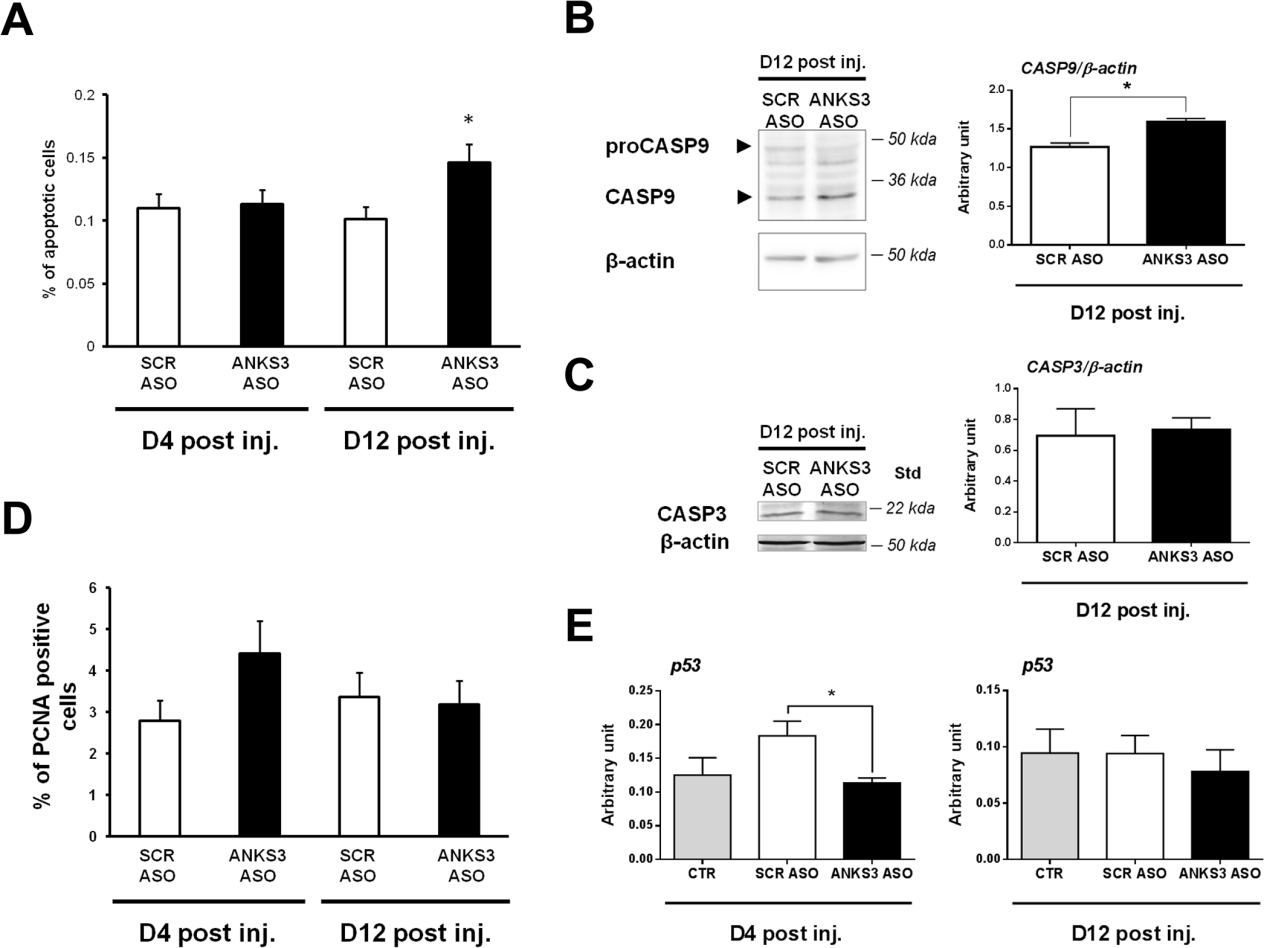
Induction of apoptosis and proliferation in *in vivo Anks3* knock-down mice. (**A**) Immunofluorescence TUNEL was used to evaluate the percentage of apoptotic cells in mouse kidneys four days (D4 post inj.) and twelve days (D12 post inj.) after the final injection of scrambled (SCR) or ANKS3 AntiSense Oligonucleotides (ASO). Apoptosis was significantly increased in SCR ASO kidneys and in ANKS3ASO kidneys 12 days after the last injection.(**B**, **C**) Abundance of caspase 9 (**B**) and caspase 3 (**C**) proteins was evaluated by Western blot twelve days after final Locked Nucleic Acid modified (LNA) ASO injection (n = 5). Protein quantification was tested in duplicate and normalized to the level of ß-actin protein. (**D**) Percentage of PCNA positive cells in kidneys from mice that received SCR and ANKS3 ASO, 4 and 12 days after the last injection. (**E**) *p53* mRNA expression was evaluated by quantitative RT-PCR four (n = 3) and twelve days (n = 5) post treatment in mice injected with LNA ASO, saline (CTR) and SCR ASO. Data are means ± SEM. Quantification of each cDNA was performed in duplicate and normalized to *Gusb* gene expression. Non-parametric Mann-Whitney U test was used to assess differences between ANKS3 ASO and control mice.*P<0.05 significantly different to control mice.

Owing to the role of many cilia proteins in cell cycle progression and proliferation and because increased apoptosis and proliferation capacity are critical early cellular events in cyst formation and PKD development in humans and mice, we investigated the role of *Anks3* on these mechanisms in mouse kidneys. The proportion of apoptotic cells was significantly higher in ANKS3 ASO treated mice twelve days after LNA ASO injection when compared to SCR ASO controls (p<0.05) (Fig 7A). Increased apoptosis in ANKS3 ASO treated mice was associated with higher renal protein abundance of the initiator caspase (caspase 9) (Fig 7B), whereas expression of the effector caspase (caspase 3) was not affected (Fig 7C). Also, the percentage of proliferating cell nuclear antigen (PCNA) positive cells was markedly increased in the kidney of ANKS3 ASO treated mice four days after the last injection, but this effect was not statistically significant (p = 0.56) (Fig 7D). In addition, *Anks3* downregulated expression was paralleled by reduced renal expression of *p53* (p = 0.013 at day 4 post LNA ASO treatment), an actor of the cell cycle arrest (Fig 7E). However, the statistical significance of this difference appeared to be due to elevated expression of *p53* in SCR ASO mice.

## Discussion

We report initial characterization of the molecular consequences of *in vivo* renal downregulated expression of mouse ANKS3, a structurally related protein partner of ANKS6 involved in renal cystogenesis in mice and rats and nephronophthisis in humans. Following experimental demonstration of both localisation of ANKS3 in the renal cilium in mice and physical interaction between the proteins *in vivo* in the mouse kidney, we show that acute LNA ASO-induced downregulation of *Anks3* expression *in vivo* in mice is associated with altered renal transcription of genes involved in the regulation of vasopressin-driven water reabsorption, cilium signalling and cell proliferation and apoptosis. These results provide novel insights into molecular mechanisms regulated by ANKS3 that may affect ANKS6function and contribute to PKD pathogenesis.

A pathological role has been originally associated to ANKS6 through the identification of the mutation R823W in its SAM domain, which causes autosomal PKD in the PKD/Mhm(*cy*/+) rat [2], and through analyses in experimental systems of the effects of mutated *Anks6* in PKD/Mhm rats [3] and in mice [6], and *Anks6* knockdown in Zebrafish [4]. Most recently, we reported spontaneous development of renal cysts in mice carrying a N-ethyl-N-nitrosourea (ENU)-induced missense mutation (I747N) located only six amino acids away from the R823W mutation in cy/+ rats, which further supports the crucial role of the SAM domain of ANKS6 in cystogenesis in mammalian species [7]. Identification of ANKS6 protein partners and the characterization of their biological role in the kidney contribute to our understanding of mechanisms involved in cystogenesis. Direct interaction of ANKS6 with the RNA binding protein BICC1 in inner medullary collecting duct cells suggests an implication of ANKS6 in BICC1-regulated cellular processes such as cell polarity, cAMP signaling or cilia orientation [9, 20–22]. Interactions between ANKS6 and nephronophthisis-associated proteins (INVS, NEK7, NEK8, NPHP3) support the role of ANKS6 in renal physiology and in cystic diseases [4, 6]. Furthermore, crystal structures of association between the proteins ANKS6 and ANKS3 suggest physical binding between their SAM domains [10], providing that they are co-expressed in the same cells, which we were able to confirm here through analysis of the mutation R823W in the ANKS6 SAM domain that prevents ANKS3-ANKS6 binding, and through evidence of their co-localisation in kidney tubules and glomeruli.

Proteins of the ANKS family are structurally related molecules characterized by the presence of both ankyrin repeats and SAM domain. The Ankyrin repeat is a 33 amino acid sequence motif which forms a helix-loop-helix-β-hairpin-loop fold and serves as a scaffold for interactions between proteins spanning a wide range of functions [23]. The SAM domain is a 70 amino acid motif present in over 1,000 proteins which mediates protein-protein interaction through formation of homo-and heterotypic oligomers [24, 25] and creates surfaces for recruiting proteins that act in downstream signaling pathway. Extensive homologies in amino acid sequence exist between ANKS1A and ANKS1B (45.8%) and between ANKS4A and ANKS4B (42%), whereas ANKS3 and ANKS6 share limited amino acid sequence conservation (16%). In spite of structural similarities between ANKS proteins, tissue specific expression has been reported, indicating that their biological functions may not be necessarily limited to pathways relevant to kidney pathophysiology. For example, ANKS1A is a scaffolding molecule in EpHA2- and EphA8-mediated signaling pathways in heart, skeletal muscle and pancreas [26], andANKS4A and ANKS4B form a scaffolding complex with Harmonin to regulate cilia hair bundle function or to facilitate signal transduction in epithelial cells [27].

The emergence of a protein network involving ANKS6, ANKS3 and BICC1 provides important clues on novel mechanisms involved in the formation of renal cysts. Association of ANKS3with ciliary abnormalities, its interaction with nephronophthisis proteins (NPHP1, NPHP4, NPHP8) and its role in cyst formation have been demonstrated following ANKS3 knockdown in zebrafish [17]. Our data demonstrate that ANKS3 is also a component of renal cilia in mice, but ANKS3 *in vivo* knock-down does not affect transcription of its interacting partners NPHP1 and NPHP4 in mice. Our results in mice also suggest that impaired osmoregulation caused by stimulation of the vasopressin and vasopressin V2 receptor systems, which are involved in cystogenesis and have led to new therapeutic developments in ADPKD [28], may at least partly account for PKD caused by *Anks6* mutation preventing ANKS6-ANKS3 interactions. The role of ANKS3 in renal vasopressin-mediated water reabsorption is suggested by results from *in vivo* downregulated expression of *Anks3* in LNA ASO treated mice which showed upregulation of renal expression of vasopressin-induced genes, such as *Vit32*, *Aqp2* and *Aqp3*. Array-based gene expression profiling in kidney of adult PKD/Mhm(*cy*/+) rats also indicated altered transcription regulation of *Aqp3* and *Aqp4* [29, 30], thus suggesting that disrupted binding between ANKS6 and ANKS3 affects vasopressin-mediated mechanisms that could be directly or indirectly involved in PKD. Furthermore, stimulated expression of renal *Aqp2* is also observed in very early PKD stages in young (10-day old) PKD/Mhm(*cy*/+) rats (Hoffmann, personal communication), suggesting that increased expression of *Aqp2* in response to experimentally-induced Anks3 downregulation may actually underlie priming molecular defects in cystogenesis. Finally, evidence of BICC1-mediated regulation of renal cAMP signaling in *Bicc1* ^-/-^ mutant mice [9] suggests a synergistic biological role of BICC1, ANKS6 and ANKS3 in regulating vasopressin/cAMP signaling processes.

Renal gene transcription profiling data in other models of PKD and in humans also support our observations and suggest that altered vasopressin-mediated mechanisms may be a consensus feature in PKD pathophysiology. For example, increased transcription of genes regulated by the cAMP responsive element binding protein 1 (CREB1), suggestive of enhanced cAMP signaling, has been reported in cystic kidneys in PKD1 patients [31]. Significantly altered expression of *Aqp1* and vasopressin-induced genes*Aqp2* and *Aqp3* was reported in PKD1^L3/L3^ mice [32]. In *Cys1* ^*cpk*^ mice, transcription of genes encoding arginine vasopressin receptor 2 and aquaporin 2 was increased in the cortical and outer medullary subsegments of the collecting duct [33]. Renal transport, vasopressin secretion and cAMP catabolic processes are mechanisms that are upregulated in PKD1 knock-out mice [34]. The role of cAMP agonists in the stimulation of cyst expansion and transepithelial fluid transport has been established in an *in vitro* model of renal cysts [35, 36]. Pharmacological EGFR inhibitor or antagonist of the vasopressin V2 receptor blocks cAMP-induced proliferation of cystic ADPKD cells and reduces cysts in rodent models of PKD [37, 38].

Genome-wide association studies (GWAS) carried out so far for common human renal diseases or renal phenotypes have not provided evidence of significant association with the locus *Anks3*. Associations between markers mapped in the vicinity of *ANKS3* and other diseases have been reported, including coronary artery disease [39] and schizophrenia [40], which broaden perspectives of applications of ANKS3 research beyond renal pathologies. In mice, *Anks3* maps close to a modifier locus of PKD progression in a cross between DBA/2-*pcy*/*pcy* and *Mus Castaneus* [41] and carries non coding polymorphisms between these strains (www.sanger.ac.uk/cgi-bin/modelorgs/mousegenomes/snps.pl), which may at least partly account for the modifying effects.

In conclusion, we have provided evidence of presence of ANKS3 in cilia in mice, co-localisation of ANKS3 and ANKS6 in mouse glomeruli and tubules and novel insights into renal molecular mechanisms affected by altered expression of ANKS3 *in vivo* in mice. Molecular consequences of *Anks3* expression downregulation *in vivo* suggest its involvement in vasopressin signaling in the kidney, which may contribute to renal cystogenesis when interactions between ANKS6 and ANKS3 are disrupted. Our data complement results from structural analyses of ANKS6-ANKS3 protein complexes and functional studies in zebrafish, which collectively contribute to building and understanding the function of a network of proteins involving ANKS6. They may uncover novel therapeutic targets for renal cystic diseases in humans, but should also contribute to understand extra-renal biological roles of ANKS6.

## Supporting Information

S1 Fig Protein sequence alignment of Ankyrin repeats region and SAM domain of ANKS3 and ANKS6. CLUSTALW2 sequence alignment was obtained by a FASTA sequence analysis algorithm (www.ebi.ac.uk/Tools/msa/clustalw2/) to compare Ankyrin repeats region (**A**) and SAM domain (**B**) in ANKS3 and ANKS6 in zebrafish, chicken, mouse, rat and human. Ankyrin repeats in ANKS6 and ANKS3 are indicated by white and black boxes, respectively. Signs in the last row mean: asterisk, identical amino acids (highlighted in black and white text); single and double dot, weakly and strongly similar amino acids (highlighted in yellow and blue), respectively. GenBank accession numbers: zebrafish ANKS3, NP_001108037.1; chicken ANKS3,XP_004945449.1; mouse ANKS3, AAH50929.1; rat ANKS3, AAH87062.1; human ANKS3, Q6ZW76.1; zebrafish ANKS6, Q5XJ13.1; chicken ANKS6,XP_004935230.1; mouse ANKS6, AAH72562.1; EMBL accession numbers: rat ANKS6, AAT76432.1; human ANKS6, Q68DC2.2.
(TIF)

S2 Fig Effect of *in vivo Anks3* knock-down in C57Bl/6 mice on body weight, organ to body weight ratio, plasma urea nitrogen and proteinuria. (**A**) Body weight follow up of mice injected with either saline (Ctr) or LNA antisens oligonucleotide scrambled (SCR ASO) or targeting *Anks3* mRNA (ANKS3 ASO). (**B**) Body Mass Index (BMI) of mice, 4 days after final injection (n = 3) and 12 days after final injection (n = 5). (**C**-**F**) Ratio of left Kidney (**C**), right Kidney (**D**), Liver (**E**) and spleen (**F**) to body weight, 4 days after final injection (n = 3) and 12 days after final injection (n = 5). (**G**) Concentration of plasma urea nitrogen in mice (n = 5 per group) twelve days after the final injection of LNA ASO. (**H**) Concentration of protein in urine of mice (n = 4 per group) twelve days after the final injection of LNA ASO. Data are means ± SEM. Mann-Whitney U test was used to assess differences between ANKS3 ASO and control mice injected with saline (Ctr) or SCR ASO. **P< 0.01 significantly different to control mice.
(TIF)

S3 Fig Renal expression of genes encoding ciliary proteins in response to *in vivo* Anks3 expression knock down. Expression of genes encoding proteins from the basal body (**A,B**), the transition zone (**C,D**) and the ciliary axoneme (**E,F**) domains of the primary cilia andfor Inversin (**G**) were quantified in kidney of the *Anks3* knock down mice. Renal transcript level of *Cep290* (**A**), *Nphp5* (**B**), *Nphp1* (**C**), *Nphp4* (**D**), *Nek8* (**E**) and *Gli2* (**F**) was evaluated by quantitative RT-PCR four (n = 3) and twelve days (n = 5) after final LNA ASO injection. Data are shown as means ± SEM. cDNA quantification was performed in duplicate and normalized to *Gusb* gene expression level. Non-parametric Mann-Whitney U test was used to assess differences between ANKS3 ASO and control mice. *P< 0.05; **P< 0.01 significantly different to control mice treated with saline (CTR) or SCR ASO.
(TIF)

S1 Table Design of the antisense oligonucleotides stabilized by Locked Nucleic Acids (LNA). Asterisks indicate phosphorothioate linkage between DNA bases. LNA bases are indicated in parentheses.
(PDF)

S2 Table ARRIVE guidelines checklist for reporting in vivo experiments in animals. (DOCX)

S3 Table Sequence of oligonucleotides used for quantitative RT-PCR. (DOCX)
