# Supplementary figures and images for: ANKS3 Co-Localises with ANKS6 in Mouse Renal Cilia and Is Associated with Vasopressin Signaling and Apoptosis In Vivo in Mice
Source: PLoS One. 2015 Sep 1;10(9):e0136781. doi: 10.1371/journal.pone.0136781 (PMC4556665; doi:10.1371/journal.pone.0136781)

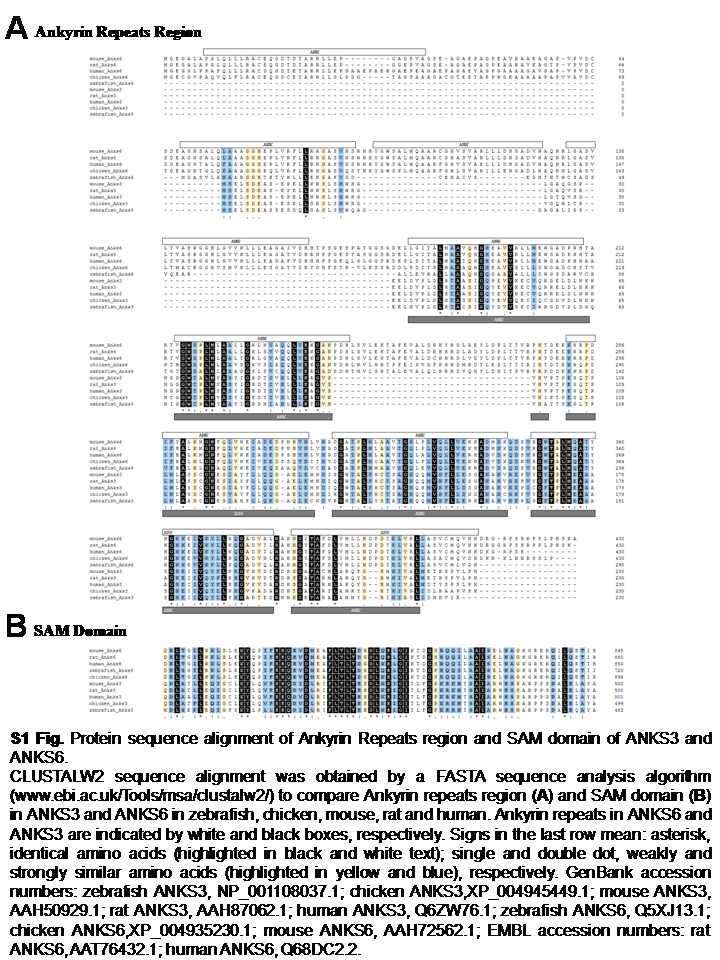

Supplement: S1 Fig — CLUSTALW2 sequence alignment was obtained by a FASTA sequence analysis algorithm (www.ebi.ac.uk/Tools/msa/clustalw2/) to compare Ankyrin repeats region (A) and SAM domain (B) in ANKS3 and ANKS6 in zebrafish, chicken, mouse, rat and human. Ankyrin repeats in ANKS6 and ANKS3 are indicated by white and black boxes, respectively. Signs in the last row mean: asterisk, identical amino acids (highlighted in black and white text); single and double dot, weakly and strongly similar amino acids (highlighted in yellow and blue), respectively. GenBank accession numbers: zebrafish ANKS3, NP_001108037.1; chicken ANKS3,XP_004945449.1; mouse ANKS3, AAH50929.1; rat ANKS3, AAH87062.1; human ANKS3, Q6ZW76.1; zebrafish ANKS6, Q5XJ13.1; chicken ANKS6,XP_004935230.1; mouse ANKS6, AAH72562.1; EMBL accession numbers: rat ANKS6, AAT76432.1; human ANKS6, Q68DC2.2. (TIF) [file pone.0136781.s001.TIF]

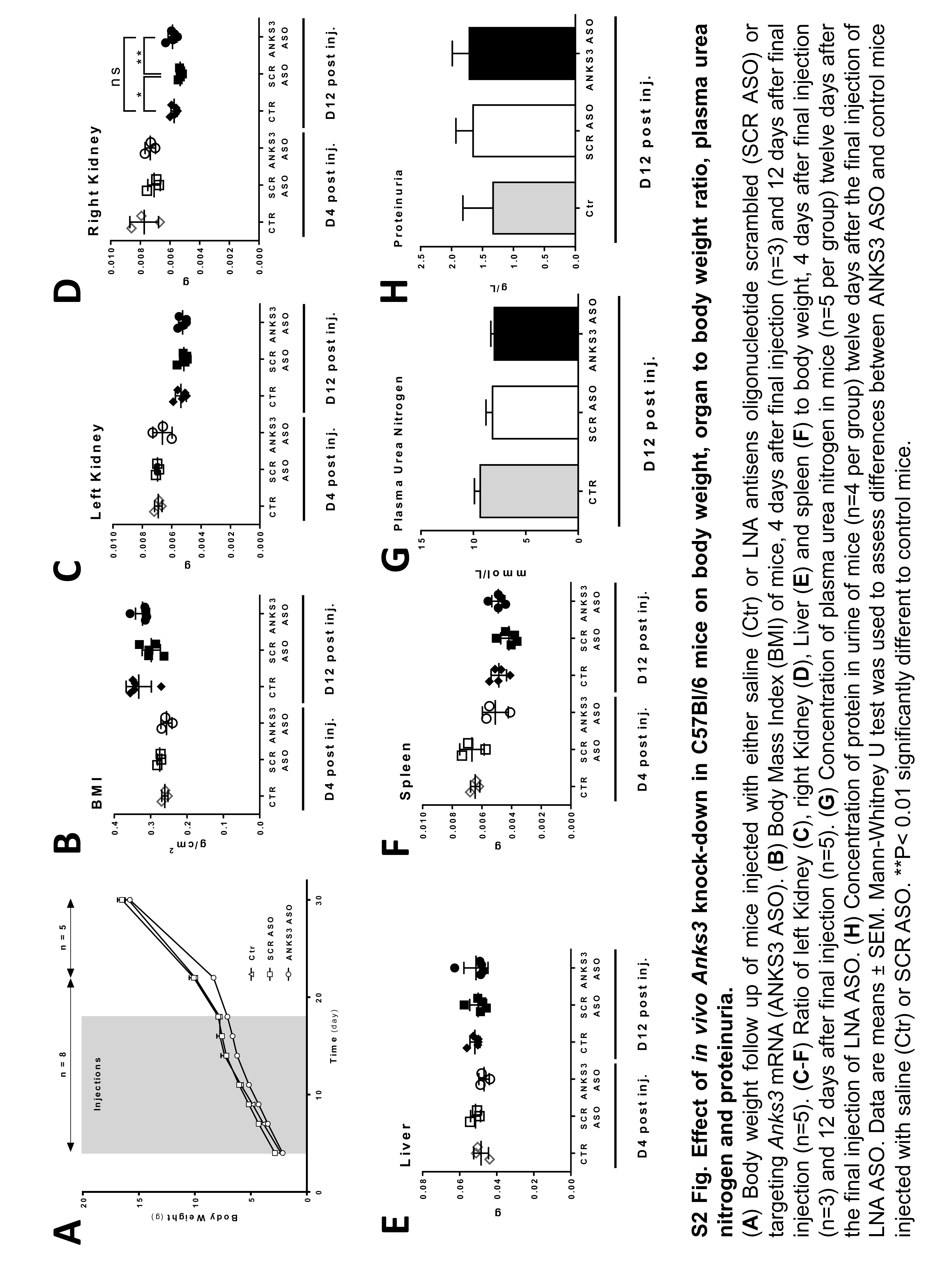

Supplement: S2 Fig — (A) Body weight follow up of mice injected with either saline (Ctr) or LNA antisens oligonucleotide scrambled (SCR ASO) or targeting Anks3 mRNA (ANKS3 ASO). (B) Body Mass Index (BMI) of mice, 4 days after final injection (n = 3) and 12 days after final injection (n = 5). (C-F) Ratio of left Kidney (C), right Kidney (D), Liver (E) and spleen (F) to body weight, 4 days after final injection (n = 3) and 12 days after final injection (n = 5). (G) Concentration of plasma urea nitrogen in mice (n = 5 per group) twelve days after the final injection of LNA ASO. (H) Concentration of protein in urine of mice (n = 4 per group) twelve days after the final injection of LNA ASO. Data are means ± SEM. Mann-Whitney U test was used to assess differences between ANKS3 ASO and control mice injected with saline (Ctr) or SCR ASO. **P< 0.01 significantly different to control mice. (TIF) [file pone.0136781.s002.tif]

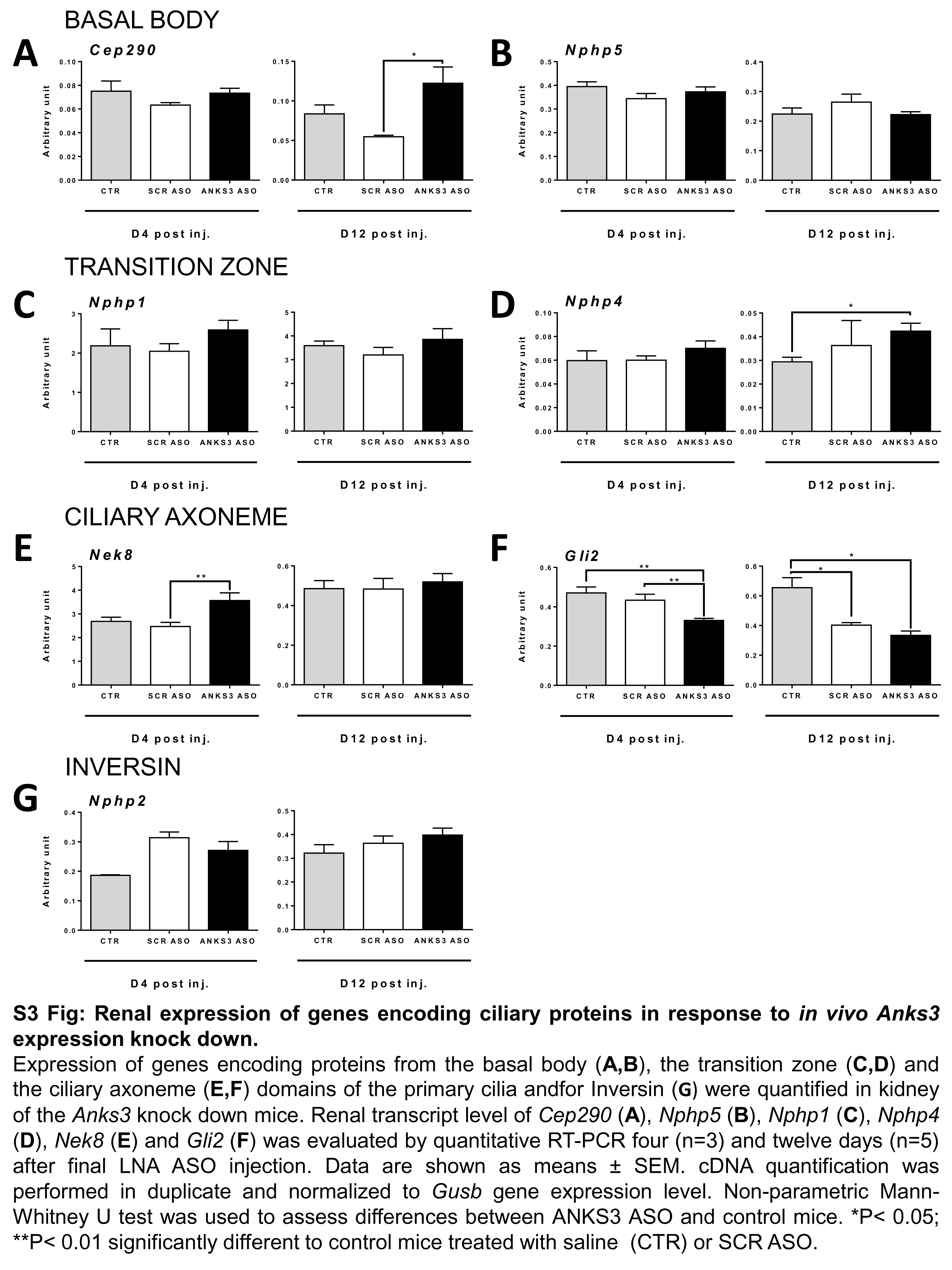

Supplement: S3 Fig — Expression of genes encoding proteins from the basal body (A,B), the transition zone (C,D) and the ciliary axoneme (E,F) domains of the primary cilia andfor Inversin (G) were quantified in kidney of the Anks3 knock down mice. Renal transcript level of Cep290 (A), Nphp5 (B), Nphp1 (C), Nphp4 (D), Nek8 (E) and Gli2 (F) was evaluated by quantitative RT-PCR four (n = 3) and twelve days (n = 5) after final LNA ASO injection. Data are shown as means ± SEM. cDNA quantification was performed in duplicate and normalized to Gusb gene expression level. Non-parametric Mann-Whitney U test was used to assess differences between ANKS3 ASO and control mice. *P< 0.05; **P< 0.01 significantly different to control mice treated with saline (CTR) or SCR ASO. (TIF) [file pone.0136781.s003.tif]
