# Supplementary material for: ANKS3 Co-Localises with ANKS6 in Mouse Renal Cilia and Is Associated with Vasopressin Signaling and Apoptosis In Vivo in Mice
Source: PLoS One. 2015 Sep 1;10(9):e0136781. doi: 10.1371/journal.pone.0136781 (PMC4556665; doi:10.1371/journal.pone.0136781)
Supplement: S1 Table — Asterisks indicate phosphorothioate linkage between DNA bases. LNA bases are indicated in parentheses. (PDF) [file pone.0136781.s004.pdf]

S1 Table. Design of the antisense oligonucleotides stabilized by Locked Nucleic Acids (LNA). Asterisks indicate phosphorothioate linkage between DNA bases. LNA bases are indicated in parentheses

**Name**	**Sequence**	**3' Modification**
ANKS3 LNA ASO_1	{CAC-AC}T*-C*C*T*-T*C*A*-C*C*A*-{CCT-CA}	Alexa Fluor 647 on Amine C7
ANKS3 LNA ASO_2	{ACA-CA}C*-T*C*C*-T*T*C*-A*C*C*-{ACC-TC}	Alexa Fluor 647 on Amine C7
ANKS3 LNA ASO_3	{CAC-AC}A*-C*T*C*-C*T*T*-C*A*C*-{CAC-CT}	Alexa Fluor 647 on Amine C7
SCR LNA ASO	{CTC-AA}C*-T*C*C*-C*A*T*-C*A*C*-{CCC-AT}	Alexa Fluor 647 on Amine C7
