# Supplementary material for: ANKS3 Co-Localises with ANKS6 in Mouse Renal Cilia and Is Associated with Vasopressin Signaling and Apoptosis In Vivo in Mice
Source: PLoS One. 2015 Sep 1;10(9):e0136781. doi: 10.1371/journal.pone.0136781 (PMC4556665; doi:10.1371/journal.pone.0136781)

**S2 Table**. ARRIVE guidelines checklist for reporting in vivo experiments in animals.

|  | ITEM | RECOMMENDATION | | Section/ Paragraph | |
| --- | --- | --- | --- | --- | --- |
| 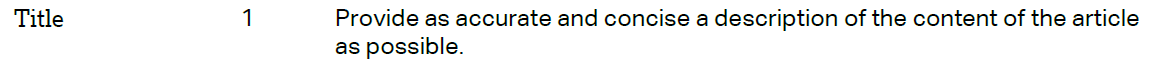 | | | Treatment of mice with a Locked Nucleic Acid (LNA) modified antisense oligonucleotides designed to inhibit the expression of ANKS3, a protein partner of a susceptibility gene for renal cyst diseases. | |  |
| 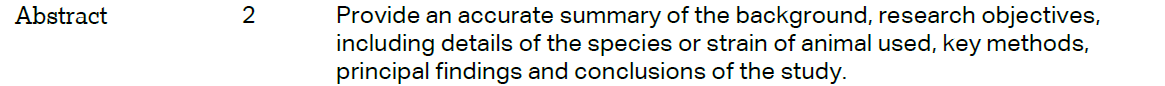 | | | **Background**: Polycystic kidney diseases and nephronophthisis are a group of several renal disorders in humans that systematically require renal transplantation in patients. Few genes responsible for these diseases are known. We identified mutations in the protein Ankyrin repeat and sterile alpha motif domain containing 6 (ANKS6) which causes renal cyst formation in the PKD/Mhm(cy/+) rat model of polycystic kidney disease and in nephronophthisis in humans.  **Research objectives and methods**: The aims of the study are to identify proteins interacting with ANKS6 using Yeast two hybrid techniques and to test their function in vivo in C57BL6/J mice treated with a Locked Nucleic Acid (LNA) modified antisense oligonucleotides designed to inhibit their expression.  **Key results**: We showed that the protein ANKS3 binds ANKS6 and is located in renal cilia. We demonstrated that binding between the two proteins occurs through their sterile alpha motif (SAM) and that the amino acid 823 in rat ANSK6 is key for this interaction. Downregulated expression of Anks3 in vivo in mice by Locked Nucleic Acid (LNA) modified antisense oligonucleotides was associated with increased transcription of vasopressin-induced genes, suggesting changes in renal water permeability, and altered transcription of genes encoding proteins involved in cilium structure, apoptosis and cell proliferation.  **Conclusions**: We provide experimental evidence of ANKS3-ANKS6 direct interaction and co-localisation in mouse renal cilia. Our results contribute to improved knowledge of the structure and function of the network of proteins interacting with ANKS6, which may represent therapeutic targets in renal cystic diseases in humans. | |  |
| INTRODUCTION | | |  | |  |
| 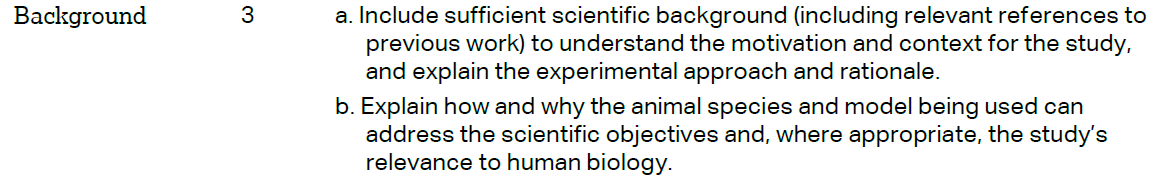 | | | 3a. Polycystic kidney disease and nephronophthisis are frequent and severe renal diseases in humans, which eventually require renal transplantation. We identified mutations in the protein ANKS6, which causes renal cyst formation in the PKD/Mhm(cy/+) rat (Brown et al. PMID: 16207829). Mutations in ANKS6 have been described in patients with nephronophthisis (Hoff et al. PMID: 23793029). It is now essential to construct the network of proteins binding ANKS6 to understand its function and mechanisms leading to cyst formation. We identified the protein ANKS3 as a protein partner of ANKS6 that requires further experiments to establish its function in kidney in a mammalian species.  3b. Renal biopsies are impossible to obtain from patients with polycystic kidney disease or nephronophthisis and from control individuals. Preliminary data on ANKS3/ANKS6 function have been obtained in zebrafish, but experiments in mouse models are essential to establish their biological roles in a mammalian species. The mouse was preferred as the volume of LNA ASO to be injected for ANKS3 knock down can be reduced, | |  |
| 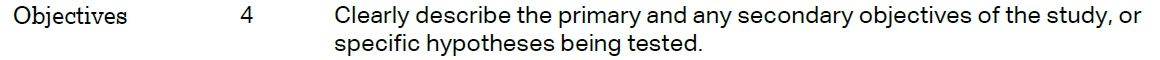 | | | Primary objective: Establish that ANSK3 binds ANKS6  Secondary objective: Provide initial characterisation of the function of ANKS3 in vivo in a mammalian species. | |  |
| METHODS | | |  | |  |
| 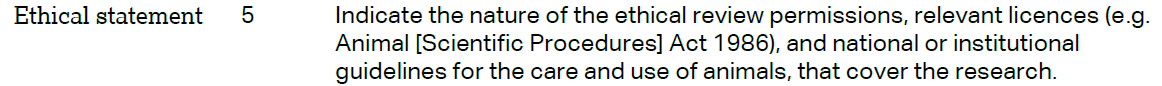 | | | Animal studies were authorized by a licence (Ce5/2012/072) under the Charles Darwin Ethics Committee in Animal Experiment, Paris, France. | |  |
| 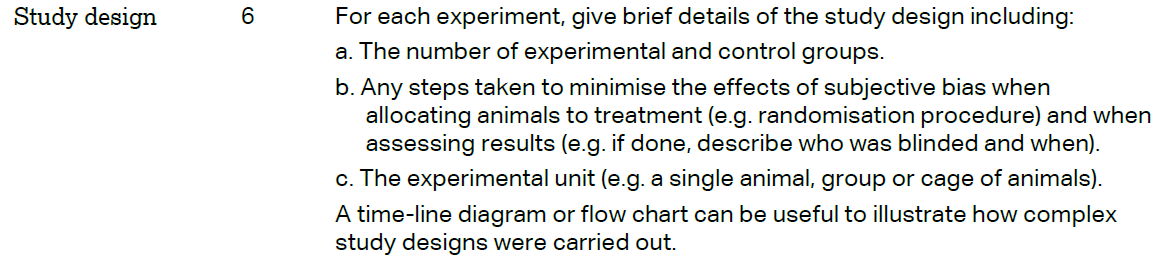 | | | 6a. One experimental group and two control groups at two time points were used (=six mouse groups): Mice injected with LNA ASO (experimental) and scrambled ASO (controls) and another group of control mice injected with saline. A total of 6 mouse groups were used (mice killed at weaning –ie. 22 days- or post-weaning at 30 days).  6b. No randomisation procedures could possibly be applied since all injections were carried out before weaning. Any attempt to randomise mice in the groups would have required transfer of newborn mice to foster mothers, which would have certainly resulted in increased risk of rejection of the newborns by the mother, reduced litter size and thus significant risk of bias in the results.  6c. Newborn/young mice were maintained with their mothers throughout the duration of the experiment until weaning. Mice of the group tested at 30 days were weaned at 22 days and maintained in groups of 8-10 mice per cage. | |  |
| 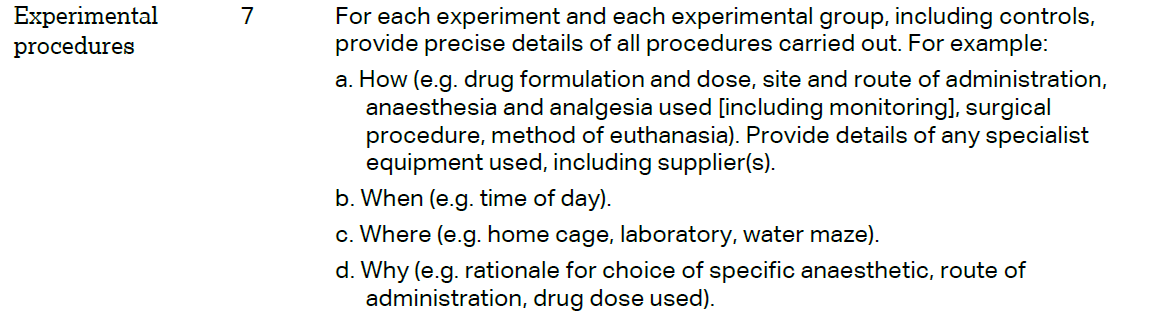 | | | 7a. Four day old mice were injected intraperitoneally with LNA ASO, scrambled ASO (300 pmol/injection) dissolved in saline o saline. Injections were repeated every other day until mice were 18 day-old.  7b. Injections were always carried out in the morning (10-11am).  7c. Mice were injected in their home cages.  7d. Intraperitoneal injection is the only way to inject substances in young (4-18days) mice. Intravenous injections are not possible in young mice without inducing major stress, and oral gavage is not appropriate for treatment with oligonucleotides. | |  |
| 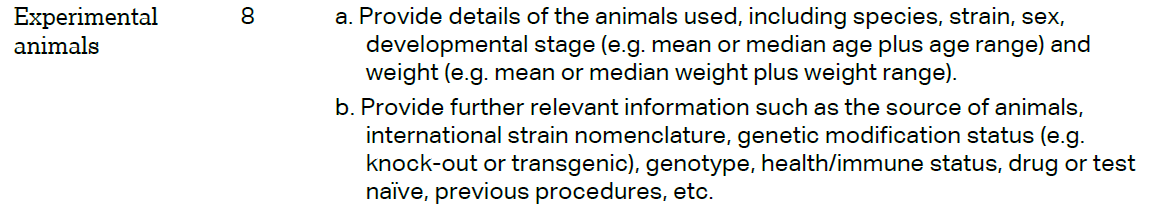 | | | 8a. Pregnant mice of the C57BL/6J strain were purchased from a commercial supplier (Janvier, Saint Berthevin, France) and delivered in the animal facility. Male and female newborn and young mice (4-18 days) were used in the experimental protocol of LNA ASO, scrambled and saline repeated injections  8b. We used standard C57BL/6J mice from the above mentioned commercial supplier. | |  |

The ARRIVE guidelines. Originally published in *PLoS Biology*, June 2010^1^

| 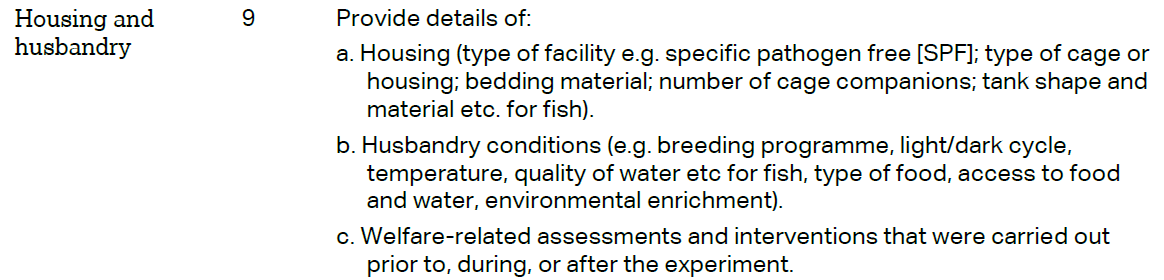 | 9a. Housing: Pregnant mice were housed individually in specific pathogen free (SPF) condition at the animal research facility at the Cordeliers Research Centre, Paris, France.  9b. Pregnant mice had free access to standard carbohydrate diet and tap water and maintained in a controlled environment (23±2°C; 12 hours light/dark cycles). No environmental enrichment was used. Newborn mice were maintained with their mothers until weaning, when they were transferred to carbohydrate diet.  9c. Health monitoring was assessed on a daily basis by observation of the animals and monitoring of body weight and food intake to detect any loss of body condition in the experimental and control groups. | |
| --- | --- | --- |
| 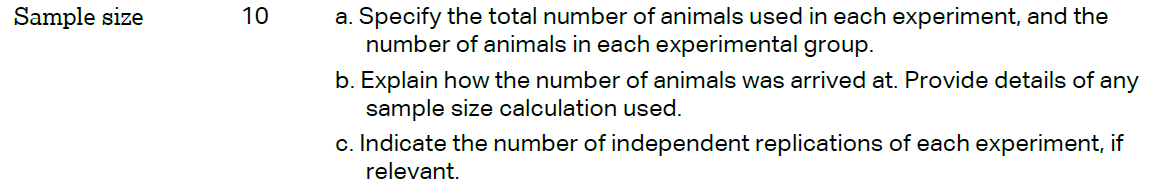 | 10a. Experiments were carried out in groups of 6 newborn mice treated with LNAASO, scrambled ASO and saline.  10b. Based on prior experience in renal physiological studies in mice, we ensured that 6 mice was the minimal number of animals to be used to minimise the effect of interindividual variability in statistical analyses.  10c. We used 6 biological replicates for each of the three experimental and control groups. | |
| 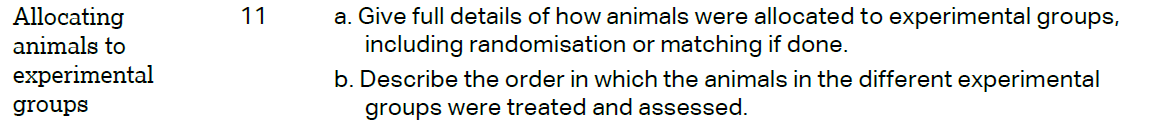 | 11a. As justified in section 6b, randomisation procedures are inappropriate for experiments carried out in young (pre-weaning) animals. Randomisation requires transfer of newborn mice to foster mothers, which would have certainly resulted in increased risk of rejection of the newborns by the mother, reduced litter size and thus significant risk of bias in the results.  11b. Mice from each litter were randomly injected every other day from day 4 until day 18. Injections were performed rapidly and manipulations of newborn and young mice were limited to avoid risks of rejection of the newborns by the mother. Mice were injected randomly as marking newborn mice (eg. marker pen) also increases the risk of rejection of the newborns by the mother. | |
| 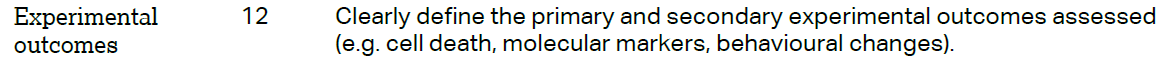 | 12. Primary outcome: Occurrence of renal cysts and changes in gene expression in sampled organs. Secondary outcomes: Analysis of physiological variables associated with changes in ANKS3 expression. | |
| 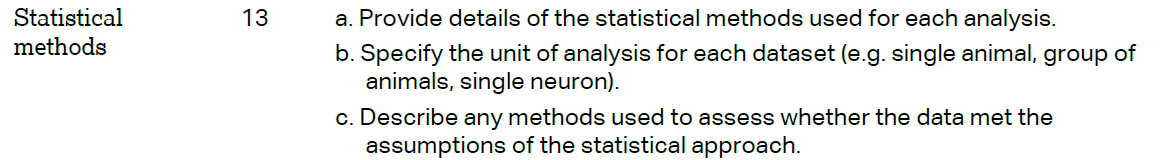 | 13a. Statistical analyses were performed with non-parametric Mann- Whitney U test or one way ANOVA test.  13b.Data from each mouse was used. Mice of the LNA ASO group were compared to the two control groups (injected with scrambled ASO or saline) in order to test the effect of ANKS3 downregulation and that of treatment with a non specific oligonucleotide.  13c. Prior to statistical analysis, normality was assessed by a Kolmogorov Smirnov test. | |
| RESULTS |  | |
| 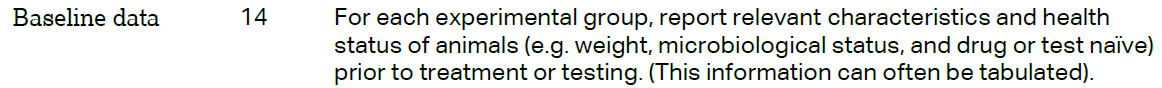 | 14. Pregnant mice were purchased from a commercial supplier, which complies with health requirements of our SPF animal facility. Mice were free viral, bacterial, and parasitic pathogens listed in our veterinary recommendations. | |
| 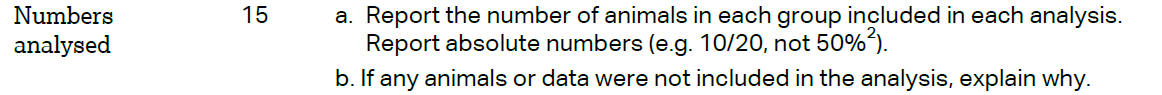 | 15a. Six mice per group were used for injection.  15b. All animals from the experimental and control groups were used in the analyses. Data may be missing for some variables due to low volume of blood or urine samples. | |
| 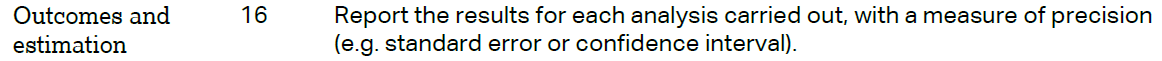 | The experimental procedure used in vivo in mice induced a significant reduction in the expression of the target gene. 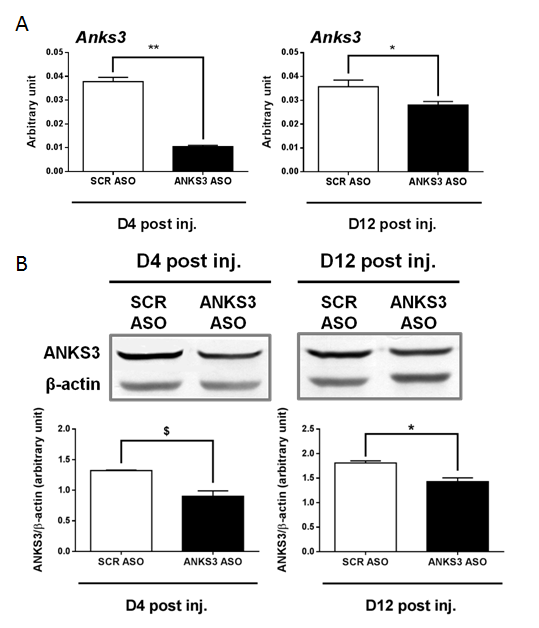 Inhibited expression of Anks3 transcript (A) and protein (B) in kidney of mice treated with LNA ANKS3 ASO when compared to mice treated with scrambled ASO. Experiments were performed four days (D4 post inj) and twelve days (D12 post inj) after the final injection. Data are mean +/- SE. *P<0.05 significantly different to SCR ASO controls | |
| 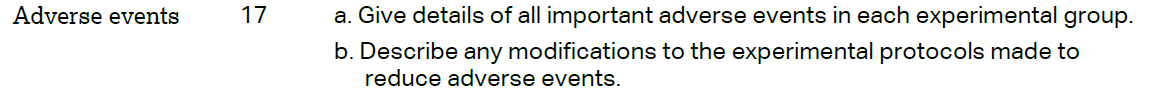 | 17a. We verified that treatment with LNA ASO and scrambled ASO in mice had no adverse effects by daily observations of the animals, measures of body weight and food intake and eventual analysis of the expression of inflammatory proteins in the kidney.  17b. Treatment of mice had no effect on body weight and food intake, and no deterioration of body condition was observed. Downregulated expression (-30%, see figure above) of ANKS3 was not associated with the occurrence of renal cysts. Adjustments of experimental protocols were therefore not required. | |
| DISCUSSION |  | |
| 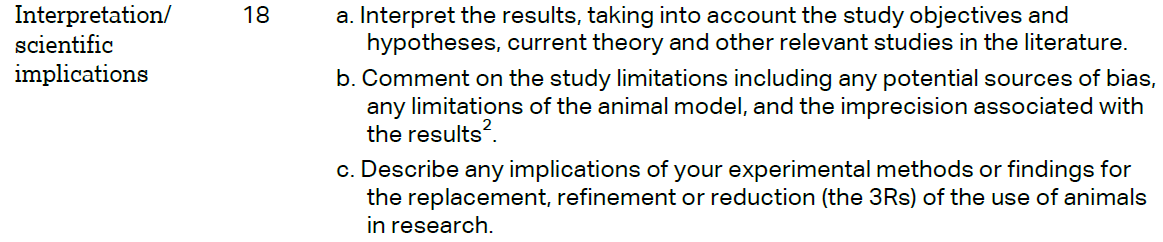 | 18a. Inhibition of ANKS3 expression in vivo provided an experimental system to test the biological role of the protein in kidney in a mammalian species. This was associated with changes in renal expression of genes known to play a role in polycystic kidney diseases and located in the renal cilium.  18b. A possible limitation of the study is that the network of proteins interacting with ANKS6 may be different in mice and in humans. Nevertheless, most of the knowledge of ANKS3/ANKS6 function is until now based in Zebrafish, and investigations of proteins involved in this network in a mammalian species are essential prior to translation to humans.  18c. We have collected a broad range of information from a limited number of animals. The main technical strength of the study lies in the possibility to knock down gene expression rather than completely inactivate the gene. Applying our method therefore results in mild phenotypic outcome and reduces mortality rate and reduces the number of animal required to achieve the goals of the study. | |
| 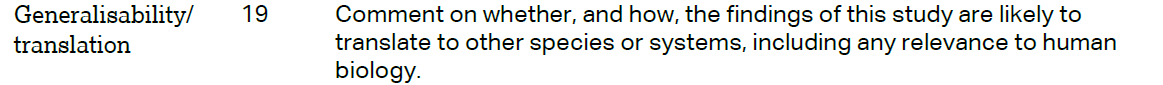 | Construction of the network of proteins interacting with ANKS6 and investigation of their function in mice are particularly important since renal biopsies cannot be collected in patients and control individuals. We know that ANKS6 and ANKS3 are expressed in human kidney and that mutations in these proteins are present in patients affected by cystic diseases. Establishing the function of the proteins in vivo in mice provide information on mechanisms involved in cyst formation that can be targeted for drug development in humans. | |
| 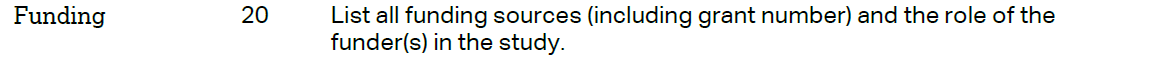 | | Work supported by grants from the Agence Nationale de la Recherche (RenalCyst, ANR-10-BLAN-1124-02), the European Community Seventh Framework Programme (FP7/2007-2013) under grant agreement N° HEALTH-F4-2010-241504 (EURATRANS), the NIH (5RO1DK100482), the Institute of Cardiometabolism and Nutrition (ICAN, ANR-10-IAHU-05) and a Wellcome Senior Fellowship in Basic Biomedical Science (057733).  No roles of funders in the design of the study. |


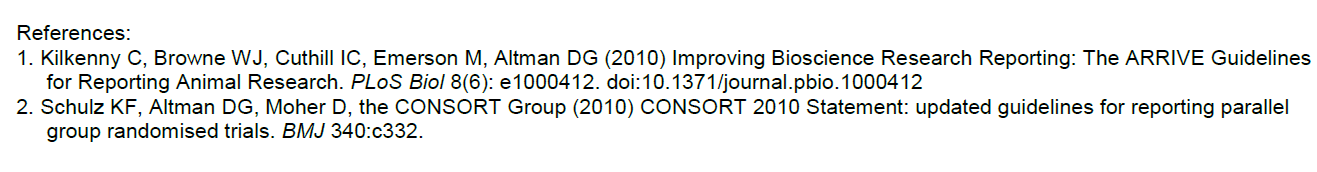

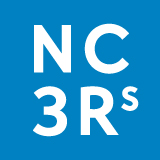

Supplement: S2 Table — (DOCX) [file pone.0136781.s005.docx]
