# Supplementary material for: ANKS3 Co-Localises with ANKS6 in Mouse Renal Cilia and Is Associated with Vasopressin Signaling and Apoptosis In Vivo in Mice
Source: PLoS One. 2015 Sep 1;10(9):e0136781. doi: 10.1371/journal.pone.0136781 (PMC4556665; doi:10.1371/journal.pone.0136781)
Supplement: S3 Table — (DOCX) [file pone.0136781.s006.docx]

S3 Table. Sequence of oligonucleotides used for quantitative RT-PCR

| Gene | Genbank | Sequences |  |
| --- | --- | --- | --- |
| *Actb* | NM_007393.3 | Forward | 5' - GACGATGCTCCCCGGGCTGTATTC - 3' |
|  |  | Reverse | 5' - TCTCTTGCTCTGGGCCTCGTCACC - 3' |
| *Anks3* | NM_028301.4 | Forward | 5' - AGGTGGTGAAGGAGTGT - 3' |
|  |  | Reverse | 5' - CAAGTGAACAATGGTATCG - 3' |
| *Anks6* | NM_001024136.1 | Forward | 5' - TCTGAGCTGAATGCGGGCA - 3' |
|  |  | Reverse | 5' - CACTGCTTTCAAAGGAAGAG - 3' |
| *Aqp1* | NM_007472.2 | Forward | 5' - CTCCCTAGTCGACAATTCAC - 3' |
|  |  | Reverse | 5' - ACAGTACCAGCTGCAGAGTG - 3' |
| *Aqp2* | NM_009699.3 | Forward | 5' - CTTCCTTCGAGCTGCCTTC - 3' |
|  |  | Reverse | 5' - CATTGTTGTGGAGAGCATTGAC - 3' |
| *Aqp3* | NM_016689.2 | Forward | 5' - TTGGTGGCTGGCCAAGTGTC - 3' |
|  |  | Reverse | 5' - GTCTGTGCCAGTGCATAGAT - 3' |
| *Cep290* | NM_146009.2 | Forward | 5' - GTCTGAGAGGGAAACTTA - 3' |
|  |  | Reverse | 5' - TCAAATCTTCAATAATAAACG - 3' |
| *Gli2* | NM_001081125.1 | Forward | 5' - TACACTGTGAGCCGCCGC - 3' |
|  |  | Reverse | 5' - TGTGAGGTTGAGCAGCCC - 3' |
| *Gusb* | NM_010368.1 | Forward | 5' - CTCTGGTGGCCTTACCTGAT - 3' |
|  |  | Reverse | 5' - CAGTTGTTGTCACCTCACCTC - 3' |
| *Icam1* | NM_010493.2 | Forward | 5' - CCCACGCTACCTCTGCTC - 3' |
|  |  | Reverse | 5' - GATGGATACCTGAGCATCACC - 3' |
| *Nek8* | NM_080849.3 | Forward | 5 '- ACACCCTGCTACATCTCCC - 3' |
|  |  | Reverse | 5' - TAAGACACAGCCCAGAGCC - 3' |
| *Nphp1* | NM_016902.3 | Forward | 5' - TCCAGAAGAGCACAGGGCA - 3' |
|  |  | Reverse | 5' - GAGACCGAAGTTTCACCAGA - 3' |
| *Nphp2* | NM_010569.4 | Forward | 5' - TACACACCCCTTGATTATGC - 3' |
|  |  | Reverse | 5' - GTTTTCCTCTTCCCGTTTTTT - 3' |
| *Nphp4* | NM_153424.2 | Forward | 5' - ACGCTGTCTGGTCTATAAGG - 3' |
|  |  | Reverse | 5' - TGAGAACTGGAACTGGATGG - 3' |
| *Nphp5* | NM_177128.4 | Forward | 5' - CAGGAGAAGATGGAGAAGAAT - 3' |
|  |  | Reverse | 5' - AGAAGAGAGAATCAGTCACAAT - 3' |
| *Trp53* | NM_001127233.1 | Forward | 5' - GGCTTATGGAAACTACTT - 3' |
|  |  | Reverse | 5' - TCCTCAACATCCTGGGG - 3' |
| *Vit32* | NM_027106.4 | Forward | 5' - CCGCCCAAACAGAACCAC - 3' |
|  |  | Reverse | 5' - GTCTGATCTGGTGGAGGTA - 3' |
